# Microbial synthesis structures organic compound composition in anaerobic digestion

**DOI:** 10.1093/ismejo/wrag036

**Published:** 2026-02-20

**Authors:** Xingsheng Yang, Bo Zhao, Kai Feng, Jie Wang, Mingqian Liu, Xi Peng, Qing He, Yanjuan Lu, Hassan Waseem, Shang Wang, Mari-Karoliina H Winkler, Joana Falcão Salles, Ye Deng

**Affiliations:** State Key Laboratory of Regional Environment and Sustainability, Research Center for Eco-Environmental Sciences, Chinese Academy of Sciences, Beijing 100085, China; College of Resources and Environment, University of Chinese Academy of Sciences, Beijing 100049, China; State Key Laboratory of Regional Environment and Sustainability, Research Center for Eco-Environmental Sciences, Chinese Academy of Sciences, Beijing 100085, China; College of Resources and Environment, University of Chinese Academy of Sciences, Beijing 100049, China; State Key Laboratory of Regional Environment and Sustainability, Research Center for Eco-Environmental Sciences, Chinese Academy of Sciences, Beijing 100085, China; College of Resources and Environment, University of Chinese Academy of Sciences, Beijing 100049, China; State Key Laboratory of Biogeology and Environmental Geology, China University of Geosciences, Beijing 100053, China; State Key Laboratory of Regional Environment and Sustainability, Research Center for Eco-Environmental Sciences, Chinese Academy of Sciences, Beijing 100085, China; College of Resources and Environment, University of Chinese Academy of Sciences, Beijing 100049, China; State Key Laboratory of Regional Environment and Sustainability, Research Center for Eco-Environmental Sciences, Chinese Academy of Sciences, Beijing 100085, China; College of Resources and Environment, University of Chinese Academy of Sciences, Beijing 100049, China; State Key Laboratory of Regional Environment and Sustainability, Research Center for Eco-Environmental Sciences, Chinese Academy of Sciences, Beijing 100085, China; Fairyland Environmental Technology Co., Ltd, Beijing 100085, China; Department of Civil and Environmental Engineering, Carleton University, 1125 Colonel By Dr, Ottawa, ON K1S 5B6, Canada; State Key Laboratory of Regional Environment and Sustainability, Research Center for Eco-Environmental Sciences, Chinese Academy of Sciences, Beijing 100085, China; Department of Civil and Environmental Engineering, University of Washington, Seattle, WA 98105, United States; Faculty of Science and Engineering, University of Groningen, Groningen, 9747AG, The Netherlands; State Key Laboratory of Regional Environment and Sustainability, Research Center for Eco-Environmental Sciences, Chinese Academy of Sciences, Beijing 100085, China; College of Resources and Environment, University of Chinese Academy of Sciences, Beijing 100049, China

**Keywords:** anaerobic digestion, dissolved organic matter, microbial metabolism, molecular biosynthesis, FT-ICR mass spectrometry

## Abstract

Anaerobic digestion (AD) is a cornerstone technology for sustainable waste treatment and renewable energy recovery, yet its complex microbe–metabolite interactions remain poorly understood. Here, we combined high-resolution molecular profiling and microbial community sequencing in a three-month study across seven full-scale digesters to resolve dissolved organic matter (DOM) and microbiome dynamics. A total of 28 925 DOM molecules, including a conserved core of 1154 metabolites, were identified. By disentangling metabolic pathways, we observed complex transformation patterns that extend beyond simple substrate breakdown. Molecules within a mass window (183.57–390.81 m/z) exhibited high persistence, strong microbial associations, and distinct transformation trajectories. Within this mass window, microbial community composition and feedstock input, together explained ~30.1%–43.4% of the observed spatiotemporal variation. In each digester, 1260–2108 molecules were closely associated with microbial metabolism, forming 7.77–24.52 microbe–metabolite associations on average. The accumulation and turnover of these microbial metabolites were strongly linked to methane production and system performance, highlighting microbial processing of DOM as a significant factor shaping microbe–metabolite interactions. This perspective emphasizes the importance of microbe–metabolite interplay in AD, providing a conceptual framework for predictive monitoring and optimization of engineered biotechnologies.

## Introduction

Anaerobic biotechnology is a crucial approach for achieving carbon neutrality, reducing greenhouse gas emissions, and promoting sustainable development by treating organic waste from food residues, agriculture, and wastewater treatment plants while recovering energy-rich compounds [1–4]. In China alone, over 120 million tons of food waste are produced annually [5]. If processed through anaerobic digestion (AD), this resource could generate an estimated 23 000 GWh of electricity and cut CO_2_ emissions by ~564 million tons [5]. Although AD has been applied for over a century [6], our understanding of its underlying microbial transformation mechanisms remains limited. Researchers are only just beginning to identify and characterize specific patterns of anaerobic microbial transformations of organic subclasses, moving beyond the traditional, broad chemical oxygen demand (COD) centered assessment of organic matter degradation [7–9].

The classical model of AD, comprising hydrolysis, acidogenesis, acetogenesis, and methanogenesis, offers a foundational framework for understanding the bioconversion of organic waste [10, 11]. Within this framework, primary monomeric substrates are fermented by bacteria into intermediates such as volatile fatty acids, alcohols, H_2_, and CO_2_, which are subsequently oxidized by syntrophic microorganisms whose metabolism is thermodynamically coupled to methanogens [10, 11]. This perspective yields a robust yet inherently low-resolution understanding of organic matter conversion, classifying substrates primarily by functional roles (e.g. primary monomer, syntrophic substrate, or direct methanogenic substrate). However, such a generalized view overlooks the complex, branched pathways of carbon and electron flow, including the identities of the molecular intermediates and the specific microorganisms that produce and consume them. This limitation underscores the need for a high-resolution, molecular-level understanding of AD processes [7, 9]. Dissolved organic matter (DOM) metabolites are now increasingly recognized not merely as passive substrates but as dynamic components that respond to spatiotemporal environmental variations, microbial interactions, and chemical processes in the surrounding medium [12–16]. Consequently, DOM has been increasingly viewed through a “metacommunity ecology” lens, a perspective that conceptualizes DOM as an evolving metabolic pool whose ecological and functional significance parallels that of the microorganisms themselves [9, 12].

Ecological theories are being adopted to investigate the diversity and trajectories of DOM molecules, such as their production, degradation, migration, and transformation, thereby expanding the boundaries of research in this field [14, 17]. Within this expanding theoretical and technological landscape, a suite of new tools, such as MetaboDirect, iDOM, and Transformation-based Organic Molecular Ecological Network Analysis (TOMENA), has emerged to dissect meta-metabolism with increasing resolution [18–20]. In complex environments, metabolite abundances fluctuate rather than follow monotonic trends due to diverse sources and consumption pathways, complicating the tracing of molecular fates and transformations. TOMENA addresses these challenges by integrating molecular network analysis with time-lagged correlation comparisons to reveal directional relationships among compounds. This allows differentiation between synthetic processes that generate larger molecules and degradative processes that decompose macromolecules into smaller units. Such differentiation is crucial for understanding the interactive roles of microorganisms and metabolites within the ecological mechanisms they co-construct. High-resolution molecular identification and a deeper grasp of ecological dynamics now point toward a key frontier: the capacity to precisely steer carbon flow and microbial composition to improve the design and operation of engineered anaerobic systems.

In this study, we conducted a longitudinal sampling campaign across AD facilities in seven cities throughout China, collecting time-series samples for more than three months. By integrating 16S rRNA gene amplicon sequencing, shotgun metagenomics, FT-ICR MS (Fourier transform ion cyclotron resonance mass spectrometry), and advanced analytical frameworks, we systematically characterized the spatiotemporal dynamics of DOM metabolites and investigated the ecological and molecular mechanisms underlying these changes. From a metacommunity ecology perspective, DOM pools can be conceptualized as structured molecular assemblages analogous to biological communities, enabling microorganisms and DOM metabolites to be jointly incorporated into a unified ecological framework. Guided by this perspective, we addressed four key questions: (i) What are the dominant molecular transformation trajectories in anaerobic digesters, and what core chemical features characterize DOM diversity? (ii) How do the composition and diversity of DOM metabolites and microbial communities vary across space and time, and do persistent core taxa and molecular pools exist? (iii) What microbe–metabolite co-occurrence patterns emerge, and which microbial and molecular groups occupy key structural positions within these networks? (iv) To what extent do microbial communities shape DOM chemical diversity, and which processes exert the strongest control over DOM composition, particularly with respect to the relative roles of microbial biosynthesis versus degradation? The overall objective of this study is to elucidate the spatiotemporal transformation patterns of DOM for enhancing the management and optimization of AD systems.

## Materials and methods

### National-level sample collection

Between June and September 2022, this study carried out sampling campaigns at seven food waste AD facilities across China. These facilities were located in seven cities spanning from north to south: Qiqihar (QQ), Beijing (BJ), Qinhuangdao (QH), Jingzhou (JZ), Changsha (CS), Wenzhou (WZ), and Foshan (FS). During the sampling period, samples were collected from the fermenters at each facility at six distinct time points. Additionally, in July, samples were also collected from the materials entering the anaerobic digesters to assess the regional influence of the original feedstock. Detailed sampling information is provided in Supplementary Tables S1 and S2, and the process flow of the AD system is shown in Fig. S1. Physicochemical characters were measured, including soluble total nitrogen (STN), ammonia nitrogen (NH_4_^+^-N), soluble chemical oxygen demand (SCOD), total solids (TS), pH, salinity, soluble carbohydrate (S-carbohydrate), soluble protein (S-protein), and biogas production. Additional methodological details and physicochemical data are provided in the Supplementary Information (SI).

### DNA extraction and metagenomic preprocessing

Solid–liquid mixed samples (six time-series samples from each of the seven facilities, *n* = 42) obtained from the anaerobic digesters were separated using 0.22 μm filters. DNA was extracted from the filter cake using the PowerSoil DNA Isolation Kit (MO BIO Laboratories, USA) according to the manufacturer’s instructions. The extracted DNA was then amplified for the V4 region of the 16S rRNA gene using the universal primers 515F and 806R, which have been shown to provide excellent coverage for both bacteria and archaea [21]. The amplification protocol and sequencing followed the methods reported in previous studies [22]. Sequencing data underwent quality control and preliminary processing on the Denglab Metagenomics Analysis Pipeline (DMAP, https://dmap.denglab.org.cn) [9, 23]. During this process, zero-radius operational taxonomic units (ZOTUs) were obtained using Unoise3 [24], and taxonomic classification was performed using the RDP Classifier [25]. High-quality genomic DNA from 42 digester samples was also subjected to paired-end (2 × 150 bp) metagenomic sequencing. After quality control and filtering, clean reads were assembled and binned using the MetaWRAP pipeline integrating MEGAHIT, MetaBAT, MaxBin, and CONCOCT [26]. High-quality bins (completeness >90%, contamination <10%) were dereplicated with dRep [27], resulting in 413 nonredundant metagenome-assembled genomes (MAGs) for downstream analyses. Taxonomic annotation of MAGs and identification of rRNA genes were performed using GTDB-Tk and barrnap, respectively [28]. Carbon metabolic capacities were inferred using DRAM [29]. Details are provided in the SI.

### Fourier transform ion cyclotron resonance mass spectrometry sample preparation and data preprocessing

FT-ICR MS was used to detect the DOM composition in both the influent substance (*n* = 7) entering the anaerobic digesters and the transformed material within the digesters (*n* = 42). The filtered liquid from the collected samples was first extracted into methanol, following the method described in a previous study [30]. The extracted samples were then analyzed using an FT-ICR MS with a 15.0 T superconducting magnet (Bruker SolariX, Bruker, USA) in negative ion mode [31]. Following a previously established workflow [9], each molecular peak was assigned a unique molecular formula after quality control and classified based on elemental composition. Detected molecules were retained within a mass range of 100–800 Da. This range included organic acids with molecular weights above that of valeric acid, which were predominantly classified as lipid-like based on elemental ratios. Amino acids such as tyrosine and aggregated oligopeptides were classified as protein-like, whereas monosaccharides and polysaccharides were grouped as carbohydrate-like. These compounds are major components of the biomass of animals, plants, and microorganisms. Additional compounds included unsaturated hydrocarbon-like, tannin-like, lignin-like, and condensed-aromatic-like molecules, most of which are of plant origin. This element-based classification provides a conceptual indication of potential molecular properties and is reported using “-like” terminology (e.g. lipid-like, carbohydrate-like) rather than representing strict correspondence to specific classes of natural organic compounds. All detected molecules were then compared with metabolites in the MetaCyc database [32] to infer their potential biochemical properties. Details are provided in the SI.

### Diversity and relative abundance analysis

We evaluated molecular diversity and compositional structure from two aspects: (i) tracking changes in DOM composition from influent to anaerobic digesters for understanding the fundamental material conversions and (ii) characterizing the spatiotemporal distribution of DOM molecules across digesters in different regions. To characterize the spatiotemporal distribution of molecules, we defined molecular occurrence at three levels: (i) occurring, referring to molecules detected at least once; (ii) ubiquitous, referring to molecules detected at least once in each region (spatial distribution); and (iii) persistent, referring to molecules detected in all samples collected over time within a region or across multiple regions (temporal distribution). All molecular composition analyses were based on relative abundances calculated from normalized FT-ICR MS peak intensities, in line with standard practice [13, 17]. Diversity analysis of microbial communities was performed following the same procedures as those applied to DOM composition.

### Molecular traits analysis

To evaluate molecular traits [9, 13, 14], a set of elemental ratios was calculated for each molecule, including H/C, O/C, N/C, and S/C. Additional molecular descriptors included the nominal oxidation state of carbon (NOSC), double bond equivalents (DBEs), and the thermodynamic carbon use efficiency (*Y*_met_). Molecular size was characterized by both the number of carbon atoms (C number) and molecular weight (m/z). The definitions and calculation methods of these molecular indices follow those described in previously published studies [9, 13, 14]. Weighted average values of these indices were computed using the relative abundance of each molecule to reflect the overall organic chemical environment of each sample [17].

### Molecular weight-dependent variation patterns analysis

Molecular dissimilarity in segments (MDS) was defined and used to evaluate spatiotemporal variation in molecular composition across different molecular weight ranges. Specifically, a sliding window approach was applied, with a window width of 40 m/z starting at 100–140 m/z and advancing in 5 m/z increments. Within each window, Bray–Curtis distances between samples were calculated to quantify dissimilarity at specific molecular weight segments. A simple linear regression model was first established to describe the relationship between segmental molecular dissimilarity and molecular weight. Based on this model, a segmented regression analysis was further performed to identify potential breakpoints that distinguish different patterns of variation [33]. Model fit was evaluated using the Akaike Information Criterion to identify the most appropriate model. To assess the influence of feedstock and microbial community composition on the molecular profiles observed within the digesters, we applied a multiple regression on matrices (MRM) approach [34]. This allowed us to quantify and compare the explanatory power of these factors across different molecular weight segments. Feedstock DOM composition was represented by the Bray–Curtis distance of influent samples, while microbial community variation was assessed using Bray–Curtis distances calculated from ZOTU profiles. Variation partitioning analysis (VPA) was subsequently conducted to determine the proportion of variance explained by each factor and their combined effects [35].

### Organic molecular transformation analysis

Molecular transformation analysis was conducted using TOMENA, a recently developed tool (Fig. S2, https://github.com/yedeng-lab/TOMENA). This method identifies potential transformation types by analyzing both correlation patterns and mass differences between molecular pairs. It uses Fisher’s exact test to determine whether a given molecular pair exhibits a quantitative relationship and whether that relationship is associated with their specific mass differences, enabling the diagnosis of putative transformation types. To infer the direction of these transformations, TOMENA calculates time-lagged correlations, orders molecules temporally, and pinpoints precursor-product relationships. This approach incorporates time-series dynamics into molecular transformation inference. Full details of the algorithm and its advantages are available in the original publication [20]. After molecular transformations were identified, molecular pairs with increased molecular weight were classified as synthesis transformations, whereas those with decreased weight were classified as degradation transformations. Pairs with undetermined direction were labeled as “uncertain.” This strategy was adopted as a pragmatic solution, given that full transformation equations were not established and the temporal variability in molecular abundances precludes the determination of strictly monotonic changes [9, 20]. Each molecule’s role (e.g. precursor or product in synthesis or degradation) was recorded, and its dominant role was assigned based on the most frequent occurrence. For each sample, we calculated synthesis and degradation indexes to independently quantify synthesis and degradation activity and to examine their relationships with operational parameters and the surrounding organic chemical environment. Details are provided in the SI.

### Microbes–dissolved organic matter co-occurrence network analysis

Bipartite co-occurrence networks were constructed to evaluate the interactions between microorganisms and DOM metabolites, with separate networks built for each of the seven regions. Network construction and analysis were performed using the iNAP pipeline (https://inap.denglab.org.cn) and corresponding locally deployed scripts [36]. Details are provided in SI. The associations between environmental factors and network properties were assessed at both the global network and module levels. For each network node, correlations with environmental variables were calculated using Spearman’s rank correlation. Mantel tests were then performed based on both correlation coefficients and node topological attributes [37, 38]. Two network-based metrics were used to characterize the interaction patterns between DOM metabolites and microorganisms: the interaction push–pull index and species specificity [39, 40]. For DOM metabolites: interaction push–pull index ranges from −1 to +1. Positive values indicate influence exerted by the metabolite on microbial taxa, while negative values reflect influence received from microbes. Larger absolute values represent stronger directional influence. Species specificity ranges from 0 to 1, with higher values indicating more specialized associations between a metabolite and microorganisms. Additionally, we introduced the weighted correlation intensity (WCI) to evaluate the interaction strength between microbes and molecules involved in synthesis or degradation transformations. For such transformations, WCI is defined as follows:


$$ WCI=\frac{\sum_{i=1}^n{\overline{C}}_i{I}_i+{\sum}_{j=1}^m{\overline{C}}_j{I}_j}{\sum_{i=1}^n{I}_i+{\sum}_{j=1}^m{I}_j} $$

where ${\overline{C}}_i$ and ${\overline{C}}_j$ represent the average interaction strength between microorganisms and precursor or product molecules, and *I_i_* and *I_j_* denote their relative intensity. More details are provided in the SI.

## Results

### Overall dissolved organic matter compositions and microbial communities in anaerobic digestion facilities

**Figure 1:**
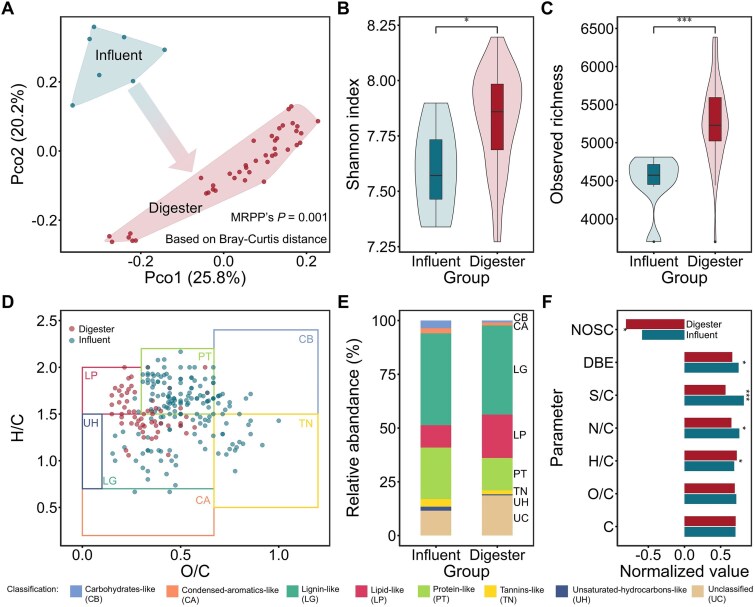
Overall DOM conversion characteristics in the anaerobic digester. We discovered the changes in DOM molecules from the influent to the digester samples. (A) Composition differences diagnosed based on Bray–Curtis distance. The pairwise difference was quantified using MRPP. (B, C) Alpha diversity, including (B) Shannon index and (C) observed richness. (D) Characteristic molecules from the two groups mapped onto a Van Krevelen diagram. These characteristic molecules were identified using the random forest method, and the most representative molecules were selected for visualization, corresponding to mean decrease Gini >0.01. (E) Comparison of the relative abundance of different classified molecules. (F) Weighted average molecular traits. Results were normalized by making the margin sum of squares equal to one. In (B), (C), and (F), statistical significance was assessed using the Wilcoxon test. The labels “*,” “**,” and “***” indicate *P*-value <.05, < .01, and <.001, respectively. Abbreviations in (D) and (F): CB, carbohydrate-like; CA, condensed-aromatic-like; LG, lignin-like; LP, lipid-like; PT, protein-like; TN, tannin-like; UH, unsaturated-hydrocarbon-like; UC, unclassified.

This study involved continuous sample collection from anaerobic digesters operating at seven facilities across China. Operating conditions and performance varied among the AD systems (Table S2). For example, methane production efficiency ranged from 0.37 ± 0.18 to 2.51 ± 0.23 m^3^/m^3^/day. To investigate the changes and conversions in the material within the AD process, we compared the DOM compositions in each facility to those of the incoming feedstock. A total of 28 925 DOM molecules were extracted from both the influent and the digesters (Fig. S3), including 11 792 in the influent and 24 443 in the reactors. A total of 7310 molecules were shared between both, representing 62.0% of the influent molecules and 29.9% of the reactor molecules. Given the high analytical resolution and reliable replicate datasets, it is unlikely that the remaining molecules were missed in the influent by chance. Therefore, a large number of molecules that appeared exclusively in the digesters can be reasonably interpreted as newly generated organic compounds formed through anaerobic biotransformation. Furthermore, we found that the molecular composition structure in the anaerobic digester underwent significant changes compared to the influent (*P* = .001, Fig. 1A), accompanied by an increase in molecular diversity (Fig. 1B and C).

To gain further insight into the molecular differences between groups, the random forest-based methods were employed to identify molecules that exhibited significant differences between the two groups (Fig. 1D). The most representative molecules were then mapped onto a Van Krevelen diagram based on the O/C and H/C ratios [41]. The characteristic molecule cluster in the influent was relatively dispersed, while the cluster in the digester was more concentrated, located in the upper-left corner of the Van Krevelen diagram, which represents higher H/C and lower O/C ratios, indicating more active components. A considerable number of molecules were identified as lipid-like molecules (20/61), with fatty acids and their corresponding molecular analogs constituting a major component of this group. Therefore, the production of lipid-like substances may be an important feature of the AD process. This is further supported by the increase in the relative abundance and diversity of lipid-like substances (Fig. 1E and Table S3). By comparison, carbohydrate-like compounds exhibited significant declines in both diversity and abundance (*P* < .05). Proteins showed a significant reduction in abundance (*P* < .05) but only a nonsignificant decrease in mean diversity. Based on the weighted average molecular traits, we found no significant changes in carbon chain length or molecular weight (Fig. 1F and Table S3). Similarly, the O/C ratio remained essentially unchanged. However, the H/C ratio increased, while DBEs and NOSC both decreased (Fig. 1F), indicating a shift in the chemical properties of the system. From the perspective of molecular oxidation, these changes suggest that the molecules became more reactive. Therefore, the consumption of nutrients such as carbohydrates by microorganisms, which support the production of diverse molecules (e.g. fatty acids) through metabolism, along with the modification of the chemical environment to make the substances more accessible for syntrophic oxidation, may be a fundamental characteristic of DOM transformation within the anaerobic digester.

To characterize the microbial communities in the anaerobic digesters and their potential roles in DOM metabolism, we obtained 1 135 050 high-quality 16S rRNA gene sequences after quality control, which were assigned to 2960 ZOTUs, comprising 2881 bacterial and 79 archaeal taxa. Each sample contained on average 479.83 ± 150.15 ZOTUs (Fig. S4). The five most abundant microbial phyla in the digesters were Bacillota, Euryarchaeota, Actinomycetota, and Bacteroidota, with mean relative abundances of 52.4%, 11.3%, 8.4%, 6.9%, and 6.8%, respectively (Fig. S5). Based on genome annotation, bacteria from Bacillota, Actinomycetota, and Bacteroidota exhibit broad metabolic capacities, including the degradation of arabinans, mixed-linkage glucans, amorphous cellulose, and xyloglucan, as well as the metabolism of fatty acids and ethanol, supporting their roles as primary fermenters and syntrophs in AD systems (Fig. S6). However, their pronounced metabolic versatility complicates efforts to determine their *in situ* functions. This highlights the importance of integrating metabolomic evidence to complement genome-based functional predictions and more accurately elucidate their ecological roles in environments. Dominant methanogens included Methanosarcinales (7.6%), a phylogenetically diverse group capable of methylotrophic, hydrogenotrophic, and acetoclastic methanogenesis, and Methanobacteriales (3.3%), which rely predominantly on hydrogenotrophic pathways. Bray–Curtis dissimilarities ranged from 24.0% to 97.9%, reflecting marked variation in community composition across samples (Fig. S7). These profiles capture a representative and functionally diverse microbiome, suggesting broad metabolic capacities that are likely to underpin DOM transformation in the digesters.

### Spatial variations in dissolved organic matter compositions across anaerobic digestion facilities

**Figure 2:**
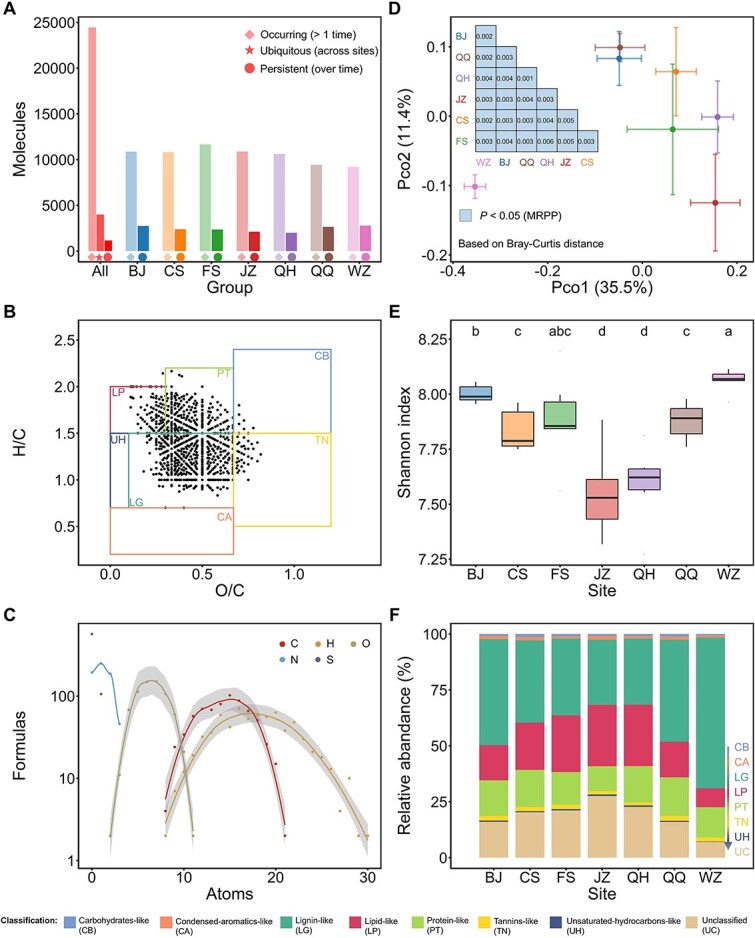
DOM composition in anaerobic digesters from different regions. (A) Counts of shared molecules in digesters. The number of molecules shared across different regions and in the overall dataset was summarized. Molecules were defined as occurring (present at least once), ubiquitous (present at least once in each region), and persistent (present in all time-series samples within a given region). (B) Core molecules identified across all samples. A total of 1154 molecules were consistently detected in all 42 samples, with the majority classified as lignin-like (673), protein-like (175), or lipid-like (100). (C) Elemental composition of lignin-like molecules identified across all samples. (D) Composition differences were diagnosed based on Bray–Curtis distance. The mean of each group is shown, along with its standard deviation, on the x- and y-axes. The pairwise difference was quantified using MRPP. (E) Alpha diversity based on the Shannon index. Different letters indicate significant differences (*P* < .05) based on the Wilcoxon test. (F) Relative abundance of molecular categories across different regions. Abbreviations: CB, carbohydrate-like; CA, condensed-aromatics-like; LG, lignin-like; LP, lipid-like; PT, protein-like; TN, tannins-like; UH, unsaturated-hydrocarbons-like; UC, unclassified.

To distinguish the cross-regional variations of DOM molecules, we further revealed the distributions and the persistence of DOM molecules in these AD engineering systems (Fig. 2A). Of the 24 443 molecules identified in the anaerobic digesters, 3980 (16.3%) were detectable across all regions. In seven facilities, the proportion of molecules consistently present across time-series samples ranged from 18.8% to 30.3%. Additionally, 1154 molecules were detectable across all regions throughout the sampling period, representing 18.1%–31.2% of the molecules detected in each sample. These molecules were mainly classified as lignin-like (673), protein-like (175), and lipid-like (100) (Fig. 2A and B). The majority of lignin-like molecules detected across all samples contained N (480/673, Fig. 2C). In contrast, only 7 of the 2960 microbial ZOTUs were detected in all samples (Fig. S8). These results confirm the presence of persistent, cross-regional organic molecules in AD systems, supporting the potential use of DOM molecular fingerprint profiles in this context, and suggesting a relatively high degree of functional redundancy among microorganisms in DOM metabolism. This inferred redundancy is further consistent with the extensive overlap in carbon metabolic functions identified at the genomic level (Fig. S6).

We observed significant regional differences in molecular composition. This was primarily supported by the significant composition differences between regions based on the Bray–Curtis distance (Fig. 2D). Moreover, there were variations in molecular diversity and the relative abundance of different molecular categories across regions (Fig. 2E and F, Table S4). For example, samples from WZ were rich in lignin-like molecules (67.4% ± 3.0%) but showed relatively low levels of protein-like (13.5% ± 1.9%), lipid-like (8.5% ± 1.0%), and carbohydrate-like (0.5% ± 0.1%) compounds. In contrast, samples from BJ, CS, QH, and QQ had higher levels of protein-like molecules, with average relative abundances ranging from 15.7% to 17.3%. Lipid-like compounds were more abundant in FS, JZ, and QH, averaging 25.3%–27.4%. CS exhibited the highest level of carbohydrate-like molecules (1.3% ± 0.3%). These results highlight that, despite having a stable core shared molecular pool, the molecular composition of the anaerobic digester still exhibits distinct regional differences.

### Molecular weight-dependent model uncovering dissolved organic matter spatiotemporal variation

**Figure 3:**
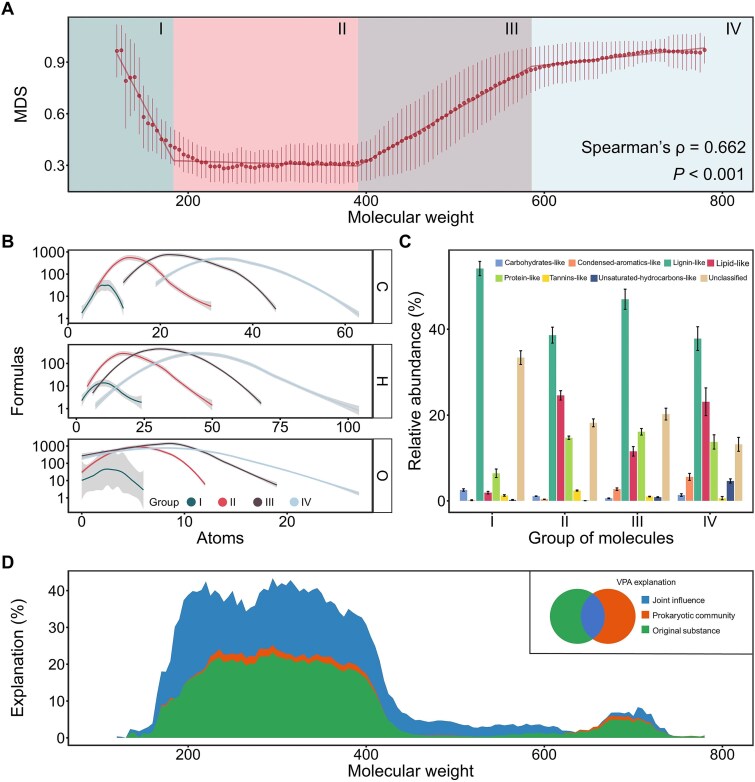
Molecular turnover patterns closely associated with molecular weight. (A) MDS in the anaerobic digester. MDS was calculated using a window size of 40 and a step size of 5, based on molecular weight and Bray–Curtis distance. The visualization shows the mean and standard deviation of the pairwise segmental dissimilarity calculated between all samples. Distinct molecular weight ranges are indicated according to breakpoint positions determined by the optimal segmented regression model (Table S5). The solid line represents the result of segmented linear fitting, which was obtained by gradually adding breakpoints to the basic simple linear fit to yield the optimized model. (B) Elemental composition of molecules in different molecular ranges. The molecular ranges were grouped based on the breakpoints from the segmented linear fit. (C) Relative abundance of molecular classifications in different molecular ranges. The visualization shows the mean and standard error. (D) Variance partitioning based on MRM applied to segmental molecular composition differences. The results were obtained across a series of molecular weight windows, with a window width of 40 and a step size of 5.

The molecular weight of organic compounds may shape the metabolic strategies applied to them by affecting their energetic yield, bioavailability, diffusivity, and transport mechanisms [20, 42]. Microorganisms may preferentially metabolize molecules within specific mass ranges, reflecting underlying metabolic trade-offs. After identifying regional differences in DOM composition within anaerobic digesters, we further explored the connection between molecular spatiotemporal variation and molecular weight, as well as the underlying mechanisms. We introduced the MDS based on Bray–Curtis distance, which assessed the spatiotemporal variations in molecular composition across different molecular weight ranges. Overall, MDS tended to increase with molecular weight (Spearman’s ρ = 0.6, *P* < .001), although an initial sharp decline was also observed (Fig. 3A). The mean MDS for each molecular weight interval has been calculated, and the segmented linear regression was applied to detect the breakpoints and characterize distinct variation regimes. Based on the optimal segmented model (Table S5), four distinct molecular weight groups were identified according to the positions of the compositional change points (Fig. 3A).

- Group I: Molecular weight 100.00–183.57. In this range, as molecular weight increases, the molecular composition difference between samples decreases rapidly.
- Group II: Molecular weight 183.57–390.81. In this range, the molecular composition remains relatively stable, with minimal differences between samples. We observed 94.4% of the previously identified persistent molecules fall within this range (Fig. S9), and 62.8% (897/1428 molecules) of the metabolites matched to the reference database in digesters also fall within this interval (Fig. S10). Together, these observations indicate that these molecules are frequently utilized by microorganisms, undergoing continuous production and consumption within the system.
- Group III: Molecular weight 390.81–585.84. In this range, the molecular composition differences between samples steadily increase as molecular weight increases.
- Group IV: Molecular weight 585.84–800.00. In this range, molecular composition differences increase slowly with rising molecular weight.

The number of identified molecules showed a unimodal relationship with molecular weight, initially increasing and then decreasing, with the maximum observed in Group III (Fig. S11). By analyzing the number of C, H, and O atoms in the molecules, we identified clear differences in elemental composition across the four molecular weight ranges (Fig. 3B). Further analysis revealed significant variations in the O/C ratio, DBE, NOSC, and *Y*_met_ across the molecular groups (Wilcoxon test, *P* < .05) (Fig. S12). These differences indicated that the molecular groups vary in redox state, stability, and bioavailability. To exclude the confounding effect of molecular weight on these indicators, the linear least-squares regressions for each metric were performed, and the residual distributions across groups were compared. This residual-based approach confirmed that the group-level differences in molecular characteristics remained significant (Wilcoxon test, *P* < .05), even after controlling for molecular weight (Fig. S12). Molecular classification patterns also varied across groups (Fig. 3C). Group I contained a high proportion of unclassified molecules (33.4% ± 10.4%). Group II was enriched in lipid-like compounds (24.6% ± 7.1%), whereas Group III showed the highest abundance of protein-like substances (16.1% ± 4.9%). Group IV was distinct for its elevated levels of condensed aromatics-like (5.6% ± 5.3%) and unsaturated hydrocarbon-like molecules (4.6% ± 3.2%). These findings highlight that, beyond molecular weight, the groups identified through MDS display considerable variation in their molecular traits and functional potential.

Regional differences and distinctive dietary practices are reflected in feedstock characteristics, while microbial communities are shaped by the source materials, inocula, and operational conditions. Both factors may further contribute to the molecular composition within anaerobic digesters. To disentangle their individual and combined effects, we applied Mantel tests, which confirmed that both microbial community composition and feedstock characteristics significantly influenced DOM composition (*P* = .001 for both, Table S6). Further analysis combining MRM with VPA revealed that microbial communities explained 16.9% of the total variance in DOM composition, whereas feedstock characteristics accounted for 31.8% (Table S6). To explore how these factors influenced spatiotemporal variation across molecular weight segments, we calculated the explained variance for each segment. The overall contribution of microbial and feedstock factors varied widely, ranging from 0.2% to 43.4% across segments (Fig. 3D). Group II molecules showed the highest total explained variance from microbial and feedstock (30.1%–43.4%), compared to Group I (0.2%–20.9%), Group III (2.7%–32.7%), and Group IV (0.4%–8.3%). The results suggest that Group II represents the most deterministically regulated subset of the DOM pool. Moreover, in 116 out of 133 molecular segments, over 50% of the explainable microbial influence on DOM composition was attributable to the joint effect of microorganisms and feedstock (Fig. 3D). Given that microbial communities are shaped by source materials and both microorganisms and feedstock are influenced by region-specific factors, further studies could build on these findings to disentangle the intrinsic drivers in greater detail.

### Decoupling degradation and biosynthesis processes in the anaerobic digestion process

In complex systems, individual molecules often participate in multiple metabolic processes, acting simultaneously as both precursors and products. The cumulative effects of these overlapping transformations influence molecular abundance dynamics, making it difficult to pinpoint specific transformation mechanisms. To overcome this challenge, we applied our newly developed approach, TOMENA, to disentangle molecular transformation patterns within AD systems [20]. TOMENA identifies potential transformation pathways by constructing molecular networks that integrate both correlation structures and molecular weight differences. It further infers the temporal order of molecular changes using time-lagged correlation analysis, allowing the determination of transformation directionality. It enables classification of molecular transformations as either degradation or synthesis and facilitates the assignment of molecules to precursor or product roles. Degradation and synthesis are defined based on molecular weight changes within paired molecular transformations, describing net molecular shifts toward lower or higher molecular weight within the DOM pool. This definition extends the conventional AD paradigm centered on stepwise molecular breakdown, providing a flux-oriented perspective on molecular dynamics.

**Figure 4:**
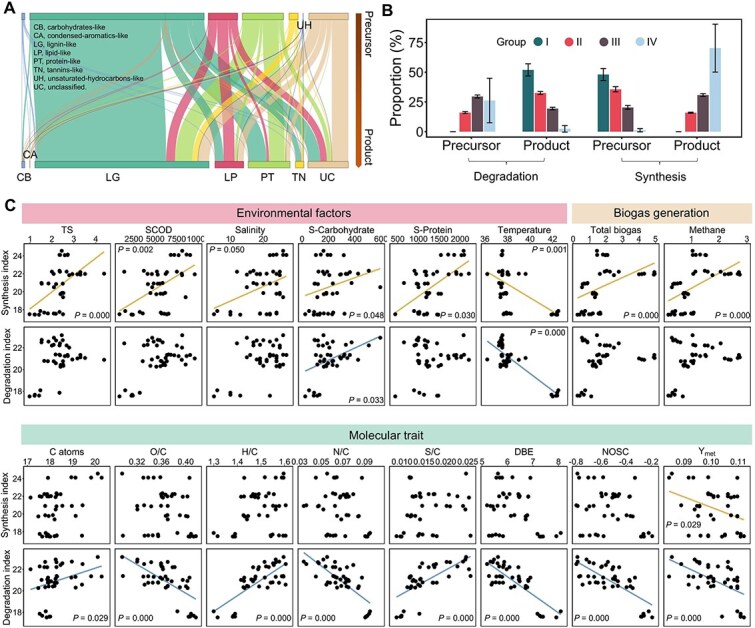
Analysis of molecular fate and the characteristics of degradation and synthesis based on molecular transformation identification. (A) Transformation associations across different molecular categories. The data are derived from the category annotations of identified molecular pairs. Visualization shows the mean values across different regions. Prior to calculating the means, resampling was performed based on the group with the smallest number of transformation pairs to ensure comparability. Abbreviations: CB, carbohydrate-like; CA, condensed-aromatic-like; LG, lignin-like; LP, lipid-like; PT, protein-like; TN, tannin-like; UH, unsaturated-hydrocarbon-like; UC, unclassified. (B) Dominant transformation identity across different molecular weight ranges. Distinct molecular weight ranges were determined by the optimal segmented regression model of MDS. For each molecule, its most frequent identity (precursor or product) across all directionally defined transformation pairs was assigned as its primary transformation role. The bar plot displays the mean and standard error across multiple regions. (C) Correlations between transformation indexes and environmental or molecular features. Synthesis and degradation indexes were calculated based on the abundance of corresponding molecular pairs in each sample. Spearman correlation coefficients were calculated to evaluate relationships between degradation/synthesis indexes and environmental variables or molecular traits. Linear regression trend lines were added to subplots where *P*-values were <.05.

Using TOMENA approach, we found that transformations involving lignin-like compounds were the most common (58.8%, based on precursor identity), followed by protein-like (13.9%) and lipid-like (9.9%) compounds (Fig. 4A). Most transformations (59.0%) occurred within the same molecular category, indicating that large-scale shifts in DOM composition are primarily driven by the cumulative, coordinated effects of multiple transformations, rather than by isolated conversion events. The ability to assign directionality to transformation pairs also allowed us to determine the dominant role (precursor or product) of each molecule. Across the seven regions, 1260–2108 molecules were predominantly involved in synthesis, while 1470–1600 molecules were primarily engaged in degradation. We found a strong link between molecular weight and dominant role (Fig. 4B). Molecules in Groups I and II were mostly synthetic precursors or degradation products, whereas those in Groups III and IV were more often synthetic products or degradation precursors. When focusing on precursor roles to reflect microbial utilization, we found that smaller molecules were more likely to act as substrates for synthesis (Fig. 4B). In contrast, larger molecules were more likely involved in degradation pathways. This dual action of synthesis and degradation appears to drive DOM composition toward an intermediate molecular weight range. As shown earlier, lignin-, protein-, and lipid-like molecules tend to cluster in Group II based on molecular weight (Table S2). The convergence of both synthesis and degradation pathways toward this group may explain its compositional stability. To evaluate the metabolic potential of each group, we quantified the number of effective transformation pairs involving individual molecules. Group II molecules exhibited the highest transformation potential, significantly higher than those in Groups I and IV, and slightly (but not significantly) higher than Group III (Fig. S13). This widespread involvement in biological reactions may be a key source of determinism in the composition of molecular metabolites.

We defined synthesis and degradation indexes based on the abundance of corresponding molecular pairs in each sample. These indices allowed us to separately evaluate the activity of degradation and synthesis in the system and explore their relationships with operational conditions and the surrounding organic chemical environment (Fig. 4C). The synthesis index showed strong correlations with operational parameters and was closely linked to biogas production. In contrast, the degradation index aligned more with the chemical characteristics of organic compounds, highlighting a functional distinction between the two processes. Only the synthesis index showed a significant association with biogas or methane production, the markers of microbial metabolic intensity. A higher synthesis index may thus serve as a proxy for elevated microbial activity. Furthermore, higher levels of TS, SCOD, salinity, S-carbohydrates, and S-proteins were positively associated with synthesis transformations (Spearman correlation, *P* < .05). These variables reflect the availability of material resources in the system, suggesting that synthesis processes are promoted under conditions of greater substrate richness. In contrast, degradation transformations were significantly associated with molecular features indicative of reactive and complex organic matter: a higher number of carbon atoms (implying larger, more complex molecules); a higher H/C ratio; and lower O/C ratio, DBE, and NOSC values (*P* < .05). These characteristics are consistent with enhanced substrate breakdown and reduced molecular recalcitrance. Some parameters, including S-carbohydrates, temperature, and *Y*_met_, were significantly correlated with both synthesis and degradation processes (*P* < .05). Among these, S-carbohydrates, as readily available energy sources, likely fuel both biosynthetic and catabolic pathways. Whereas the optimal temperature for mesophilic AD is around 35°C [43, 44], elevated temperatures (e.g. 42.25°C ± 0.24°C in WZ) appeared to negatively impact both synthesis and degradation activities. As a proxy for microbial carbon use efficiency, lower *Y*_met_ indicates organic matter that is more readily utilized by microbes, thus supporting both synthesis and degradation processes. These shared factors, linked to energy availability and thermodynamic feasibility, highlight key constraints that shape microbial metabolism in anaerobic digesters.

### Molecular ecology network between microorganisms and dissolved organic matter metabolites

**Figure 5:**
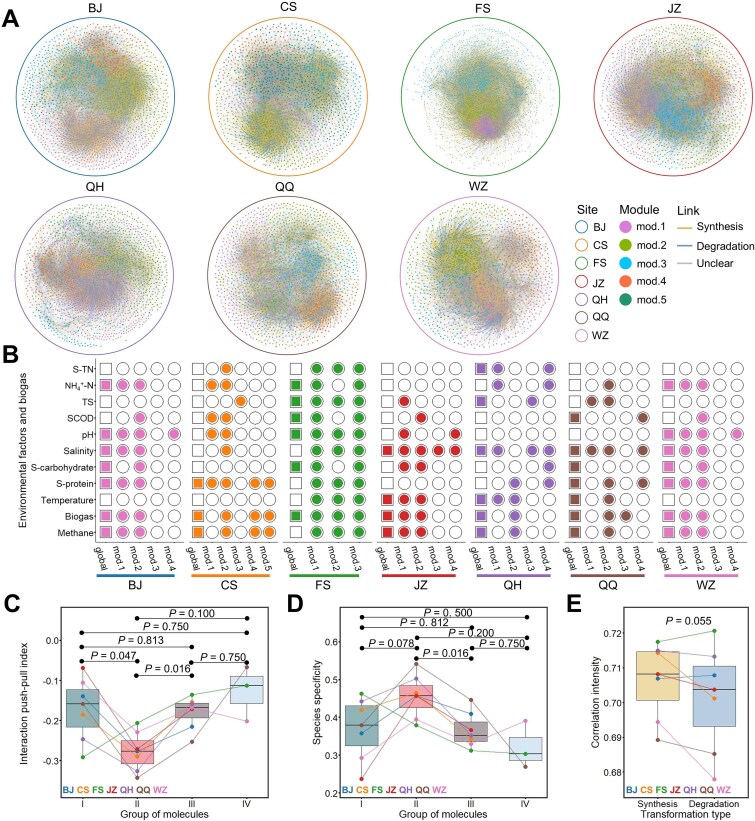
Bipartite co-occurrence network analysis between microbes and DOM metabolites. (A) Bipartite networks were constructed separately for samples from each city. (B) Associations between environmental factors and the network traits of both the global network and different modules were evaluated using Mantel tests based on Spearman correlations. Module IDs are assigned independently for each network and are not comparable across networks. Solid symbols indicate statistically significant correlations (*P* < .05). (C) Interaction push–pull index and (D) species specificity of molecules across different molecular weight ranges. Distinct molecular weight ranges were determined by the optimal segmented regression model of MDS. Each point represents the mean value for a sample within a given group. Pairwise significance was assessed using the Wilcoxon test with paired comparisons. Due to the high variability of molecules in Group IV and the requirement that molecules appear at least five times across six time points for network construction, some cities lacked molecules retained within this range. (E) Interaction strength between microbes and DOM metabolites primarily involved in degradation or synthesis. DOM metabolites were classified into degradation or synthesis groups based on their previously identified dominant role. Pairwise comparisons were evaluated using the Wilcoxon test with paired comparisons.

Molecular ecological network analysis provides a viable framework for integrating microorganisms and metabolites within a metacommunity ecology perspective, enabling the characterization of the collective features of microbially mediated metabolism and the identification of key ecological roles that structure the entire system. Using samples from seven cities, we built city-specific microbial–DOM bipartite networks (Fig. 5A and Table S7). Within these AD systems, microorganisms and DOM molecules formed complex interaction patterns over time. Individual networks consisted of 155–272 microbial nodes and 1418–3092 molecular nodes, with average node degrees ranging from 7.138 to 19.348 (Table S7). In six of the seven regions, both nestedness and weighted nestedness were significantly lower than those observed in 100 randomized rewired networks (*P* < .001). This suggests that the microbial–DOM bipartite networks exhibit nonrandom, non-nested structures. Each network was partitioned into three to five distinct modules (mod.1 to mod.5) based on internode connectivity. Key roles of microbes and molecules in the networks were identified based on their contributions to within-module connectivity and cross-module linkages (Fig. S14). The topological roles of microorganisms and molecules were assigned according to their relative contributions to within-module connectivity and cross-module linkages. All nodes identified as network hubs or module hubs were microorganisms; after removing redundancy, these comprised 15 network hubs and 319 module hubs, respectively. In contrast, 89.9% of connector nodes linking different modules were molecular nodes, totaling 444 compounds. These connector molecules likely mediate metabolite exchange among functionally distinct microbial guilds across modules. Based on sequence similarity, microbial hub taxa were further linked to MAGs (Fig. S15). Among these, two representative genomes, MAG153 and MAG326, exhibited contrasting metabolic potentials. MAG153 encoded pathways for carbohydrate metabolism but lacked complete fatty acid metabolic pathways, whereas MAG326 was primarily associated with fatty acid metabolism, highlighting functional differentiation among microbial hubs within the network.

The associations between environmental variables, biogas production, and both global and module-level network structures revealed regional differences in the key factors modulating microbe-DOM interactions across AD systems, reflecting operational variability between sites (Fig. 5B). Some variables, such as STN and NH_4_^+^-N, were tied to specific modules but not the overall network. In contrast, biogas production consistently influenced the entire network and was significantly linked to at least one module in every case, with *P*-values <.05. This highlights biogas production and related synthetic processes as a central driver of microbe–DOM dynamics. The interaction push–pull index was calculated to evaluate the strength and direction of associations between microbes and DOM molecules. Negative values indicate that molecules are more strongly influenced by microbial activity, with the absolute value reflecting the intensity of this influence. Among the four molecular ranges, molecules in the most stable range (Group II) showed the strongest microbial influence, exhibiting the most negative average interaction push–pull index across regions (mean = −0.277). In comparison, Groups I, III, and IV had less negative mean values (−0.171, −0.179, and − 0.127, respectively), with statistically significant differences observed for Group II versus Groups I and III (*P* = .0047 and .016), and a marginal difference versus Group IV (*P* = .100, Fig. 5C). Molecules in Group II also exhibited higher species specificity, with an average value of 0.457, compared to 0.370, 0.365, and 0.321 for Groups I, III, and IV, respectively (*P* = .078, .016, and .200, Fig. 5D), indicating more specialized associations with microbial taxa. Furthermore, using molecular identities from the TOMENA framework (Fig. 4C), we quantified interaction strengths between microbes and metabolites involved in synthesis versus degradation using the WCI metric (Fig. 5E). Across the seven regions, mean microbe–metabolite associations ranged from 7.77 to 24.52 for predominantly synthetic molecules and from 8.28 to 20.35 for predominantly degradative molecules. In most cases, molecules involved in synthesis showed stronger associations with microbes, exhibiting a slightly higher mean WCI (0.706) compared to those involved in degradation (mean WCI = 0.701), with a marginal statistical difference (*P* = .055). These findings demonstrate that AD is not solely characterized by stepwise degradation of small molecules but also involves active microbial synthesis of larger compounds. Moreover, the formation of larger molecules was more strongly linked to microbial community composition and abundance than degradative processes.

## Discussion

**Figure 6:**
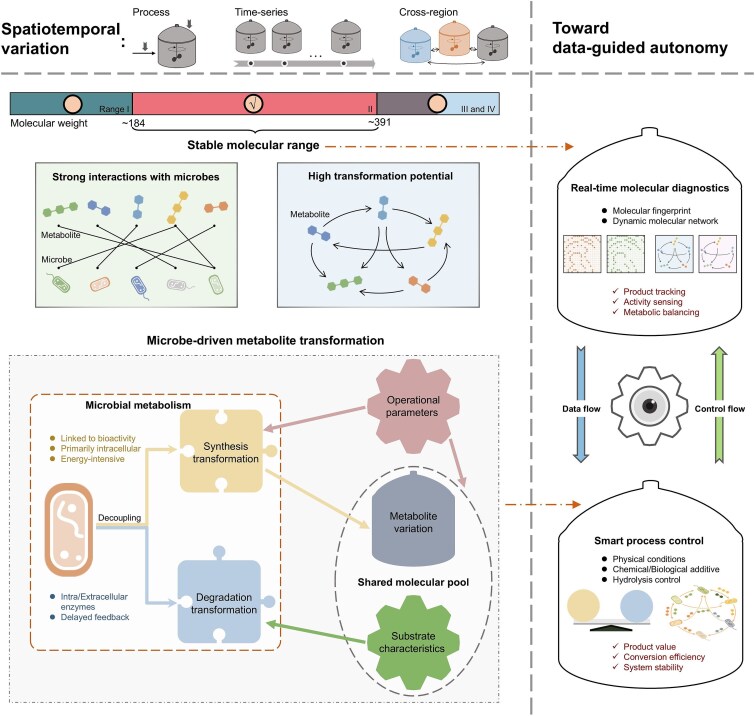
Fine-grained molecular transformation rules inform next-generation anaerobic digestion strategies. Spatiotemporal analysis of DOM variation revealed distinct transformation modes across molecular weight segments, while enabling the disentanglement of synthesis and degradation dynamics at fine resolution. These insights lay the foundation for real-time molecular diagnostics and precision metabolic control, offering a pathway to transform AD from empirical operation to targeted, adaptive bioprocessing. An era of adaptive operation, driven by online molecular data, is on the horizon.

This study investigated the spatiotemporal variability of DOM molecules and microbes in AD facilities across seven cities, expanding beyond prior research that has primarily focused on either spatial variation or limited operational changes [7, 8]. The performance disparities among facilities highlight the need for a deeper understanding of anaerobic organic metabolism to improve energy conversion efficiency. The pronounced spatiotemporal heterogeneity in DOM composition provides a valuable model for probing fundamental principles of microbial metabolism under anaerobic conditions. By emphasizing temporal changes in molecular composition, this study enables the reconstruction of molecular transformation histories and the inference of precursor–product relationships. Leveraging the newly developed TOMENA approach, segmental dissimilarity analysis, and bipartite molecular ecological network, we pushed the investigation to a finer resolution, across segmented molecular weight ranges, decoupled transformation types, and specific microbe–metabolite pairs. This framework provides new insights into molecular transformation patterns and their ecological and biochemical significance, thereby advancing both the mechanistic understanding and the applied potential of microbial DOM processing in engineered systems (Fig. 6).

Anaerobic microbial metabolism follows general principles. Despite the considerable molecular complexity and pronounced compositional variation of DOM among AD facilities, a core set of 1154 molecules persisted across all regions and sampling times. This persistent molecular set points to the existence of a metabolically stable backbone within AD systems. Comparing DOM composition before and after digestion revealed clear evidence of molecular variation, characterized by increased molecular reactivity, a shift toward more reduced states, and decreased unsaturation. These changes were exemplified by the apparent production of lipid-like compounds, reflecting active microbial transformation processes. DOM transformations were closely linked to microbial composition and metabolism, as evidenced by significant associations with spatiotemporal variation and the formation of structured, nonrandom interaction networks with DOM molecules. These findings are consistent with a previous study suggesting that microbial communities can exert deterministic effects on DOM molecular composition [9] not only in laboratory bioreactors but also in operational AD facilities. The identification of universal patterns in anaerobic microbial metabolism highlights the feasibility and importance of strategically guiding microbial processes to enhance system performance and achieve environmental and engineering objectives.

Deterministically regulated molecules exhibit clear mass ranges. Previous studies have suggested that molecular transformation patterns are closely linked to molecular size, as this influences overall energy content or transport efficiency [45, 46]. By defining and calculating MDS, we dissected the relationship between molecular spatiotemporal variation and molecular weight. This analysis identified four molecular weight segments with distinct variation patterns, moving beyond prior findings that primarily described simple linear correlations between molecular weight and observed features. We pinpointed the range of 183.57–390.81 m/z as the principal range where deterministic forces govern molecular composition. The previously identified core molecular pool was also predominantly located within this range (Fig. S9). Consistent with this pattern, metabolites that could be matched to reference metabolite databases were largely distributed within the same molecular weight window (Fig. S10). These compounds included amino acids and fatty acids, while molecules annotated as lignin-like were likely lignin monomers. Given their relatively low molecular weight, molecular transport and bioavailability may represent important factors shaping their metabolic fate. However, molecules matched to known metabolite databases accounted for only ~10% of all detected DOM compounds. This observation highlights a substantial gap in our current understanding of metabolic activity at the molecular level. Whereas our system-level analysis expands the boundaries of existing knowledge, further validation through cultivation-based experiments and the expansion of reference metabolite datasets will be essential to establish a more comprehensive foundation for interpreting DOM metabolism in AD systems.

Deterministic molecular range exhibited frequent and strong interactions with microbial communities. By examining the molecular features of molecular Group II, characterized by low variability and high explained variance, we found that molecules in this range (183.57–390.81 m/z) exhibited tighter interactions with microbes. This was evident in a higher degree of microbial contribution to molecular variation and stronger microbial “push” within interaction networks (Figs 3D and 5C). These results suggest that microbial activity supports both the persistence and broad distribution of molecules in the deterministic molecular range. These DOM molecules also showed high metabolic reactivity (Fig. S13), implying their participation in diverse microbial metabolic pathways [8, 46]. They may function as key intermediates in microbial metabolism and cross-feeding, such as serving as exchangeable substrates between metabolically complementary microbial partners [47, 48]. Molecules in Group II also demonstrated greater species specificity, indicating that they are likely subject to selective drivers by microbes, thereby being actively produced, exchanged, acquired, and utilized [14, 49]. These characteristics, frequent utilization and intentional anabolism, may thus form the foundation of the deterministic regulation of the underlying microbial populations and metabolite pool.

Decoupling degradation and synthesis advances the mechanistic understanding of anaerobic microbial metabolism. In complex ecosystems, unlike controlled experimental setups without external carbon supplementation, organic molecules are simultaneously produced and consumed [50]. This results in observed DOM abundances reflecting the superimposed effects of coupled transformation processes, complicating efforts to trace molecular fates [8, 13]. By leveraging time-series data, we were able to disentangle these overlapping dynamics and resolve the distinct temporal trajectories of synthesis and degradation. Our findings first demonstrated a clear distinction between degradation and synthesis processes within the system and subsequently revealed a clear functional separation between these processes. Synthesis, defined as net molecular transformations toward higher molecular weight, was more tightly coupled to operational parameters and represents an energy-intensive microbial activity central to sustaining metabolic turnover. Degradation, by contrast, was relatively unregulated, showing weak associations with microbial activity levels and instead is driven largely by substrate characteristics, particularly biodegradability. This distinction underscores the need to treat synthesis and degradation as independent processes when analyzing and optimizing AD systems.

Microbial synthesis metabolism is the primary force shaping the composition of DOM metabolites. In our study, close associations between microbes and DOM primarily originated from synthesis metabolism, which requires active energy investment to support microbial functions [20]. This reinforces the hypothesis that biosynthesis is a central process through which microbes shape DOM composition. Synthesis activity was also tracked using system-level indicators of microbial activity, such as biogas production, and thus matched the structure of microbial–DOM interaction networks. From an ecological and energetic perspective, microorganisms may actively synthesize larger organic molecules to accumulate biomass, thereby increasing the size of the metabolically active microbial population and enhancing the system’s overall metabolic capacity. The capacity of microbial communities to invest energy in biosynthesis reflects a high level of metabolic activity and efficient energy acquisition, which, in turn, can support thermodynamically constrained processes such as methanogenesis through tightly coupled community interactions. Conventional views of AD have largely been built around stepwise degradation processes. Our findings extend this framework by demonstrating that microorganisms simultaneously synthesize larger organic molecules, thereby contributing to DOM construction. From a metacommunity ecology perspective, biosynthesis of larger molecules, owing to its tight coupling with both microbial composition and metabolite dynamics, represents a critical focus for understanding the joint assembly of microbial communities and DOM pools. This conceptual framework may be broadly applicable to understanding the distribution and transformation of organic matter across diverse ecosystems. Furthermore, given the positive association between biosynthetic activity and methanogenesis, strategies that modulate thermodynamic conditions, substrate availability, and community structure to promote efficient microbial synthesis may offer a pathway to enhance energy recovery in engineered systems such as anaerobic digesters. These inferences are currently derived from correlative and statistical analyses. Given the energetic constraints of methanogenesis, it remains unclear whether synthesis-associated DOM patterns directly promote methane production or whether both reflect shared underlying drivers, such as substrate availability. Accordingly, these interpretations should be treated with caution. Future experimental validation, for example, through isotope tracing to resolve carbon fluxes into biosynthetic versus degradative molecular pools, will be essential for rigorously testing these hypotheses and advancing a mechanistic understanding of microbial construction and transformation of DOM.

Deterministic molecular range and synthesis–degradation decoupling framework offer transformative potential for AD operations. By developing real-time molecular diagnostics focused on the 184–391 m/z domain, operators could move beyond reactive troubleshooting toward predictive, metabolically informed system management. Practically, feasible strategies may include identifying the structural backbones of the microbe–metabolite interaction network based on the topological roles of molecules or microorganisms, such as network cores, module hubs, and connector nodes that link the entire system (e.g. as illustrated in Fig. S14). Complementary approaches could further involve *in silico* random node removal to evaluate losses in network connectivity or efficiency, thereby assessing system robustness and identifying critical molecular or microbial control points. Streamlined network-based monitoring strategies, such as tracking a targeted subset of ~50 keystone molecules or molecule–microbe pairs, may enable early detection of microbial dysbiosis and shifts in syntrophic interactions, allowing timely intervention or maintenance before process destabilization. Ultimately, integrating these molecular insights with automated dosing systems for synthesis-supporting additives could increase biogas yields while minimizing the risks of over-hydrolysis. Such advancements have the potential to reposition AD facilities from basic waste processors to precision-engineered biofactories, capable of delivering high-efficiency energy recovery and controlled biochemical conversion.

## Supplementary Material

supporting_information_wrag036

## Data Availability

Raw sequencing data were submitted to the Genome Sequence Archive in the National Genomics Data Center under accession number CRA027678 and CRA029320. The main scripts for data analysis and the tables of detected molecules, resampled ASV, and potential transformations are available via Github (https://github.com/yedeng-lab/Microbial_metabolism_during_anaerobic_digestion/tree/Seven-AD-facilities).
