# Supplementary material for: Microbial synthesis structures organic compound composition in anaerobic digestion
Source: ISME J. 2026 Feb 20;20(1):wrag036. doi: 10.1093/ismejo/wrag036 (PMC12978654; doi:10.1093/ismejo/wrag036)
Supplement: supporting_information_wrag036 [file supporting_information_wrag036.pdf]

Microbial synthesis structures organic compound composition in anaerobic digestion

Xingsheng Yang [1,2], Bo Zhao [1,2], Kai Feng [1,2], Jie Wang [3], Mingqian Liu [1,2], Xi Peng [1,2], Qing He [1], Yanjuan Lu [4], Hassan Waseem [5], Shang Wang [1], Mari-Karoliina H. Winkler [6], Joana Falcão Salles [7], Ye Deng [1,2,*]

[1] State Key Laboratory of Regional Environment and Sustainability, Research Center for Eco-Environmental Sciences, Chinese Academy of Sciences, Beijing 100085, China

[2] University of Chinese Academy of Sciences, Beijing 100049, China

[3] State Key Laboratory of Biogeology and Environmental Geology, China University of Geosciences, Beijing 100053, China

[4] Fairyland Environmental Technology Co., Ltd, Beijing 100085, China

[5] Department of Civil and Environmental Engineering, Carleton University, 1125 Colonel By Dr, Ottawa, ON K1S 5B6, Canada

[6] Department of Civil and Environmental Engineering, University of Washington, Seattle, WA 98105, USA

[7] Faculty of Science and Engineering, University of Groningen, Groningen 9747AG, Netherlands

* Corresponding authors.

E-mail address: yedeng@rcees.ac.cn (Ye Deng).

Supplementary Texts

National-level sample collection

From June to September 2022, this study carried out sampling campaigns at seven food waste anaerobic digestion facilities across China. These facilities were located in seven cities spanning from north to south: Qiqihar (QQ), Beijing (BJ), Qinhuangdao (QH), Jingzhou (JZ), Changsha (CS), Wenzhou (WZ), and Foshan (FS). The food waste used for anaerobic digestion originated from households and restaurants within the service areas, collected daily through decentralized waste collection and centralized treatment facilities. China's dietary culture is deeply rooted in its vast geography, where diverse climates, terrains, and ecosystems shape the availability of ingredients and regional food preferences [1]. Typical Chinese meals are rich in carbohydrates, with rice, noodles, and steamed buns as staples, complemented by proteins from pork, poultry, seafood, and tofu. Common cooking methods such as stir-frying, steaming, stewing, and roasting produce a wide variety of food residues. Regional characteristics strongly influence the composition of food waste. BJ and QH, influenced by northern Shandong-style cuisine, generate high-fat, high-protein waste from wheat-based staples and meat dishes. QQ (Northeast cuisine) produces meat- and oil-rich waste with moderate salt. WZ (Zhejiang cuisine) features moist, seafood-dominated waste with low grease. CS (Hunan cuisine) yields oil- and spice-rich waste with noticeable acidity, reflecting bold, chili-heavy flavors. FS (Cantonese cuisine) generates moist, balanced waste from light, steamed dishes and soups. JZ (Hubei cuisine), located along the Yangtze River, combines rice, wheat, and freshwater fish, resulting in a mixed nutrient profile.

The process flow of the anaerobic digestion system is shown in Fig. S1. Facility volumes ranged from 2,154 m^3 to 9,500 m^3. Methane production efficiency ranged from 0.37 to 2.51 m^3/m^3/day. During the sampling period, samples were collected from the fermenters at each facility at six distinct time points. Additionally, feedstock samples were taken from the materials entering the anaerobic digesters, with the July samples specifically used to assess the regional influence of the original feedstock. Detailed sampling information is provided in Supplementary Tables S1 and S2. All sampling was

conducted between 10:00 and 14:00, when the facilities were operating under the same underlying socio-economic patterns and were hence relatively stable [2]. Efforts were made to collect samples during the summer and early autumn to mitigate the potential seasonal effects on microbial and molecular composition. The substantial geographical range of the sampling locations facilitates the identification of universal patterns in material turnover and the impact of geographical separation on the system's characteristics.

Analysis of physicochemical properties

Each sample (influent, n = 42; digester, n = 42) was vacuum-filtered using 0.22 μm filters for physicochemical analysis and 0.45 μm filters for mass spectrometry. Physicochemical properties were measured according to a previous study [3]. Briefly, soluble total nitrogen (STN), ammonia nitrogen (NH_4^+-N), and soluble chemical oxygen demand (SCOD) were quantified using reagent tubes (HACH, USA). Total solids (TS) were determined using standard gravimetric methods, while pH and salinity were measured using calibrated meters. In addition, soluble carbohydrate (S-carbohydrate) and soluble protein (S-protein) were detected based on the anthrone-sulfuric acid method [4] and the bicinchoninic acid assay [5], respectively. Biogas production was monitored using online meters installed at each facility. Total gas and methane outputs were calculated based on daily volumetric generation.

Metagenomic preprocessing

Solid-liquid mixed samples (six time-series samples from each of the seven facilities) obtained from the anaerobic digesters were separated using 0.22 μm filters. DNA was extracted from the filter cake using the PowerSoil™ DNA Isolation® Kit (MO BIO Laboratories, USA) according to the manufacturer's instructions. The extracted DNA was then amplified for the V4 region of the 16S rRNA gene using the universal primers 515F (5'-GTGYCAGCMGCCGCGGTAA-3') and 806R (5'-GGACTACHVGGG TWTCTAAT-3'), which have been shown to provide excellent coverage for both bacteria and archaea [2]. The amplification protocol and sequencing followed the methods reported in previous studies [6]. Purified products were quantified using Nanodrop 2000 spectrophotometer (Nanodrop Technologies, USA) and then pooled in

equimolar ratio for library construction. Sequencing was finally performed on an Illumina NovaSeq platform (Biozeron Biotechnology Co., Ltd, Shanghai, China). Sequencing data underwent quality control and preliminary processing on the Denglab Metagenomics Analysis Pipeline (DMAP, https://dmap.denglab.org.cn) [7, 8]. After the quality control procedure consistent with our previous study [9], the zero-radius operational taxonomic units (ZOTUs) were generated using UNOISE3 with a minimum abundance of 8 and a mapping similarity of 0.97 [10]. Taxonomic annotation was performed using the Ribosomal Database Project classifier based on RDP training set No.19 [11].

High-quality genomic DNA extracted from 42 anaerobic digester samples was also subjected to paired-end (2 × 150 bp) metagenomic sequencing on the Illumina NovaSeq platform (Biozeron Biotechnology Co., Ltd., Shanghai, China). Each sample produced at least 10 GB of raw sequence data. Duplicate reads were first removed using FastUniq v1.1 [12]. Adapter sequences and low-quality regions were then trimmed with Trimmomatic v0.39 using the parameters SLIDINGWINDOW:4:20 LEADING:3 TRAILING:3 MINLEN:50 ILLUMINACLIP:TruSeq2-PE.fa:2:30:10:1:true [13]. The quality of the processed reads was assessed by FastQC v0.11.7 and summarized across all samples using MultiQC v1.16 [14]. Metagenomic assembly was performed with MetaWRAP v1.2.1 using the MEGAHIT assembler [15]. The assembled contigs were subsequently binned using MetaWRAP, which integrates three binning algorithms including MetaBAT, MaxBin, and CONCOCT. The resulting bins were refined using the bin_refinement module based on genome completeness (>90%) and contamination (<10%) thresholds [15]. Bins fulfilling these criteria were retained as high-quality metagenome-assembled genomes (MAGs). To remove redundancy across all samples, the nonredundant MAG set was generated using dRep v3.5.0 [16], yielding 413 non-redundant MAGs for downstream analyses. Taxonomic annotation and rRNA gene identification for individual MAGs were performed using GTDB-Tk v1.5.0 [17] and barrnap v0.9 (https://github.com/tseemann/barrnap), respectively. Carbon metabolic capacities, including functions related to carbohydrate-active enzymes (CAZy), short-chain fatty acid (SCFA) metabolism, methanogenesis, and methanotrophy, were

annotated using DRAM [18, 19]. The 16S rRNA gene sequences recovered from MAGs were then aligned against representative ZOTU sequences derived from amplicon sequencing using BLAST. For each ZOTU, only the highest-scoring match was retained.

FT-ICR MS sample preparation and data preprocessing

Fourier transform ion cyclotron resonance mass spectrometry (FT-ICR MS) was used to detect the dissolved organic matter (DOM) composition in both the influent substance (n = 7) entering the anaerobic digesters and the transformed material within the digesters (n = 42). The filtered liquid from the collected samples was first extracted into methanol, following the method described in a previous study [20]. The extracted samples were then analyzed using an FT-ICR MS with a 15.0 T superconducting magnet (Bruker SolariX, Bruker, USA) in negative ion mode [21]. To ensure the accuracy of the instrument's measurements, a set of natural organic compounds was used for calibration [20]. Additionally, results from 300 scans were accumulated to obtain the final mass spectrometry data [22]. After the quality control process, a list of peaks with a signal-to-noise ratio > 5 and mass measurement error < 0.5 ppm was generated. Within the molecular elemental composition range of $C_{1-100}H_{1-200}O_{0-50}N_{0-5}S_{0-3}$, and molecular weight in the range of 100-800 Da, molecular formulas were assigned to each peak using Data Analysis software v4.2. Formulas that did not conform to the elemental ratios typically found in natural organic compounds were excluded based on the following criteria: $H \leqslant 2C + 2 + N$, $0.3 \leqslant H/C \leqslant 2.5$, $O/C \leqslant 1.3$, $N/C \leqslant 0.67$, $O + N + S \leqslant C$, and $S \leqslant O$ [7, 23]. The molecular formula with the simplest heteroatom count (N + S) and smallest mass measurement error was then retained for each molecular peak [22]. The detected molecules were classified as lignin-like, lipid-like, unsaturated-hydrocarbon-like, condensed-aromatic-like, protein-like, carbohydrate-like, and tannin-like components and unclassified components based on their elemental composition [7].

Further comparison was performed between the detected DOM metabolites and the MetaCyc metabolite database based on elemental composition to establish closer associations with microbial metabolism. The MetaCyc dataset v29.0 contains 20,072 chemical compounds [24]. Only compounds with defined molecular formulas

composed solely of C, H, O, N, P, and S elements were retained, as these represent the molecular types typically detected by FT-ICR MS. For each metabolite, elemental composition and charge information were obtained using its InChI identifier. To ensure comparability with FT-ICR MS data, all metabolites were neutralized by adjusting hydrogen ion counts according to their charge states, followed by manual verification. This process yielded a dataset containing 17,897 metabolites, corresponding to 9,699 unique molecular formulas. During formula-based matching, each detected molecular formula in this study was allowed to correspond to multiple potential metabolites in the MetaCyc database. The matching procedure was implemented using a locally executed script.

Organic molecular transformation analysis

Molecular transformation analysis was conducted using TOMENA, a recently developed tool (Fig. S2, https://github.com/yedeng-lab/TOMENA). Briefly, a Fisher's exact test was applied to evaluate whether element-based molecular difference between compound pairs were statistically associated with their abundance correlations, thereby identifying potential molecular transformation types. To further infer transformation directionality, time-lagged correlations were calculated by shifting one molecular time series by a single time point. The direction of transformation between paired molecules was then determined based on the time lag at which the maximum correlation occurred. The transformation analysis was conducted separately for each region. To ensure robustness, only molecules detected in at least five out of six samples were included. Molecular pairs were evaluated using the Maximal Information Coefficient (MIC), with only those exceeding an MIC threshold of 0.99 retained for further analysis [25]. Furthermore, detection of specific mass differences as potential transformation types was restricted to cases with at least 200 valid molecular pairs. For each molecular pair with a potential transformation relationship, time-lagged correlations based on MIC were calculated to infer directionality [26]. Pairs showing an increase in molecular weight were classified as synthesis transformations, while those showing a decrease were classified as degradation transformations. Pairs with undetermined direction were labeled as 'uncertain'. Each molecule's role in these transformations (e.g., precursor or

product in synthesis or degradation) was recorded, and its dominant role was assigned based on the most frequent occurrence. For each sample, we defined and calculated Synthesis and Degradation indexes to independently assess the levels of synthesis and degradation activity in the system and to explore their relationships with operational conditions and the surrounding organic chemical environment. These indices were based on the relative abundance of molecular pairs classified as synthesis or degradation, and were calculated as follows:

$$Synthesis\ index = \frac{N_S}{N_S + N_D + N_U}$$

$$Degradation\ index = \frac{N_D}{N_S + N_D + N_U}$$

where N_S, N_D and N_U represent the number of molecular pairs identified as synthesis, degradation, and uncertain transformations, respectively. Additionally, the number of valid molecular pairs involving each molecule in a transformation relationship was used to represent its metabolic reactivity potential within the system [27].

Microbes-DOM co-occurrence network analysis

Bipartite co-occurrence networks were constructed to evaluate the interactions between microorganisms and DOM metabolites, with separate networks built for each of the seven regions. Network construction and analysis were performed using the iNAP pipeline (https://inap.denglab.org.cn) and corresponding locally deployed scripts [28]. Only microorganisms and molecules detected in at least five out of six samples per region were included in the analysis. All other parameters were set to the recommended default values. Specifically, correlations were calculated using the SparCC method to ensure robust interaction detection, and only pairwise relationships with a correlation coefficient (r) $\geqslant$ 0.6 and a significance (P) < 0.05 were retained [29]. Network modularity was evaluated using a simulated annealing algorithm. After module identification, we calculated the within-module connectivity (Zi) and among-module connectivity (Pi) to assign network roles to individual nodes [30]. Nodes were classified as follows: (i) Peripheral nodes (Zi $\leqslant$ 2.5, Pi $\leqslant$ 0.62), (ii) Connector hubs (Zi $\leqslant$ 2.5, Pi > 0.62), (iii) Module hubs (Zi > 2.5, Pi $\leqslant$ 0.62), and (iv) Network hubs (Zi > 2.5, Pi > 0.62). To assess the significance of the observed structure, 100 randomized

networks were generated by rewiring links while preserving node degree distributions [31].

Supplementary Tables

Table S1 Sampling times and locations. This study was conducted from June to September 2022.

**Sampling city**	**Longitude and latitude**	**Abbreviation**	**Volume** (m^3)	**Temperature** (°C)	**Sampling date** (Digester, N=6)	**Sampling date** (Influent)
Qiqihar	124.02° E, 47.17° N	QQ	3200	36.52–38.05	15-Jun, 27-Jun, 18-Jul, 01-Aug, 15-Aug, 13-Sep	18-Jul
Beijing	116.11° E, 40.05° N	BJ	3000	36.20–39.60	13-Jun, 04-Jul, 18-Jul, 01-Aug, 15-Aug, 19-Sep	18-Jul
Qinhuangdao	119.63° E, 40.00° N	QH	3200	37.29–38.30	15-Jun, 29-Jul, 14-Jul, 20-Jun, 03-Aug, 17-Aug	14-Jul
Jingzhou	112.35° E, 30.33° N	JZ	7500	37.26–37.52	17-Jun, 01-Jul, 15-Jul, 29-Jul, 19-Aug, 16-Sep	15-Jul
Changsha	113.03° E, 28.26° N	CS	9500	37.50–37.63	17-Jun, 01-Jul, 15-Jul, 25-Aug, 08-Sep, 17-Sep	15-Jul
Wenzhou	120.63° E, 27.51° N	WZ	2154	37.90–43.50	16-Jun, 14-Jul, 28-Jul, 11-Aug, 25-Aug, 15-Sep	14-Jul
Foshan	113.00° E, 23.00° N	FS	5200	37.47–37.53	17-Jun, 02-Jul, 16-Jul, 29-Jul, 19-Aug, 16-Sep	16-Jul

Table S2 Environmental variables and substance characteristics measured across anaerobic digestion facilities. Statistical differences among facilities were evaluated using the Wilcoxon test. To indicate statistical significance, different letters were assigned to the results. Groups that do not share any letters are significantly different from each other ($p < 0.05$), while groups that share at least one letter are not significantly different from each other.

**Factors**		**BJ**	**CS**	**FS**	**JZ**	**QH**	**QQ**	**WZ**
Influent	STN (mg/L)	1933.33 ± 1338.16 bcd	2533.33 ± 962.64 bc	2566.67 ± 564.51 b	2300.00 ± 758.95 bcd	1583.33 ± 636.92 cd	1400.00 ± 469.04 d	6750.00 ± 3317.08 a
	NH_4^+-N (mg/L)	278.33 ± 68.53 cd	365.00 ± 93.75 bc	1110.00 ± 382.67 a	258.33 ± 77.57 cd	433.33 ± 71.46 b	220.00 ± 86.72 d	310.00 ± 141.84 bcd
	TS (%)	9.50 ± 0.71 a	7.38 ± 0.73 bcd	5.84 ± 1.35 d	7.93 ± 0.64 b	7.66 ± 1.57 bc	6.76 ± 0.91 cd	8.89 ± 1.43 ab
	SCOD (mg/L)	66933.33 ± 7978.64 a	50416.67 ± 12713.52 bc	37416.67 ± 10246.84 c	43850.00 ± 8029.38 c	41250.00 ± 14023.23 bc	52300.00 ± 11390.35 b	42100.00 ± 11057.31 bc
	pH	3.98 ± 0.12 b	3.98 ± 0.20 b	5.38 ± 1.04 a	4.03 ± 0.35 b	5.31 ± 0.82 a	4.02 ± 0.18 b	4.55 ± 0.13 a
	Salinity (‰)	53.17 ± 8.47 a	39.17 ± 8.77 ab	30.67 ± 8.36 b	38.17 ± 8.70 b	31.50 ± 10.91 b	39.83 ± 5.91 b	40.00 ± 7.04 ab
	S-Carbohydrate (mg/L)	17319.77 ± 4362.39 a	1324.54 ± 847.62 c	2279.13 ± 2647.11 c	4788.85 ± 3090.33 b	1412.60 ± 993.37 c	4546.46 ± 3709.45 bc	7150.15 ± 4330.78 b
	S-Protein (mg/L)	9823.61 ± 11458.02 ab	4963.83 ± 5080.74 abc	2289.31 ± 452.72 c	4365.75 ± 1352.94 b	2397.86 ± 546.44 c	3543.33 ± 813.14 b	7664.89 ± 2753.01 a

Factors		BJ	CS	FS	JZ	QH	QQ	WZ
Digester	STN (mg/L)	1733.33 ± 564.51 c	2566.67 ± 1141.34 bc	3850.00 ± 622.09 a	3966.67 ± 1764.84 ab	2483.33 ± 658.53 bc	2533.33 ± 859.46 bc	5933.33 ± 2630.34 a
	NH_4^+-N (mg/L)	850.00 ± 296.11 c	1383.33 ± 555.33 bc	2418.33 ± 153.55 a	1813.33 ± 788.41 ab	1395.00 ± 439.12 bc	1528.33 ± 362.85 b	1200.00 ± 839.83 bc
	TS (%)	3.43 ± 0.50 a	2.38 ± 0.31 bc	2.53 ± 0.17 b	1.95 ± 0.38 c	2.07 ± 0.30 c	2.45 ± 0.06 b	1.44 ± 0.48 d
	SCOD (mg/L)	6686.67 ± 1845.94 ab	4995.00 ± 2514.72 b	7921.67 ± 855.14 a	5041.67 ± 627.99 b	5641.67 ± 1085.18 b	5955.00 ± 1401.47 b	2076.67 ± 815.44 c
	pH	7.55 ± 0.06 c	7.74 ± 0.07 b	7.91 ± 0.08 a	7.74 ± 0.06 b	7.79 ± 0.12 ab	7.79 ± 0.05 b	7.83 ± 0.15 ab
	Salinity (‰)	18.83 ± 3.31 b	20.17 ± 5.15 ab	24.67 ± 1.97 a	19.33 ± 3.33 b	21.50 ± 2.26 b	24.17 ± 2.14 a	11.00 ± 6.13 c
	S-Carbohydrate (mg/L)	314.16 ± 86.77 a	253.20 ± 142.89 ab	189.38 ± 73.58 b	252.74 ± 199.05 ab	214.39 ± 64.99 ab	191.19 ± 52.31 b	84.07 ± 39.96 c
	S-Protein (mg/L)	1856.56 ± 291.87 a	1135.33 ± 196.44 c	1888.06 ± 226.53 a	996.02 ± 198.18 c	1071.24 ± 104.38 c	1315.83 ± 155.07 b	1069.93 ± 385.39 bc
	Temperature (°C)	38.15 ± 1.16 bcd	37.49 ± 0.12 cd	37.50 ± 0.03 c	37.42 ± 0.09 d	37.94 ± 0.26 b	37.66 ± 0.24 bcd	42.25 ± 0.24 a
	Biogas ($m^3/m^3/d$)	4.55 ± 0.43 a	2.18 ± 0.41 b	1.80 ± 0.28 bc	0.70 ± 0.14 e	1.43 ± 0.28 cd	1.30 ± 0.09 d	0.61 ± 0.29 e
	Methane (m3/m3/d)	2.51 ± 0.23 a	1.52 ± 0.37 b	1.27 ± 0.22 b	0.42 ± 0.11 d	0.93 ± 0.19 c	0.78 ± 0.07 c	0.37 ± 0.18 d

**Factors**		**BJ**	**CS**	**FS**	**JZ**	**QH**	**QQ**	**WZ**
Removal efficiency	TS (%)	63.67 ± 6.72 c	67.34 ± 6.33 c	54.96 ± 9.29 d	75.53 ± 3.64 b	71.54 ± 10.09 b	63.19 ± 5.20 cd	83.27 ± 6.31 a
	SCOD (%)	89.61 ± 4.23 b	88.70 ± 8.58 ab	77.74 ± 5.23 c	88.06 ± 3.27 b	85.48 ± 3.65 b	87.30 ± 7.67 b	94.87 ± 2.52 a

Table S3 Diversity, relative abundance, and weighted molecular weight of molecules in different categories in the influent and the digesters. Statistical significance was assessed using the Wilcoxon test.

Category	Observed richness			Relative abundance (%)			Weighted molecular weight		
	**Influent**	**Digester**	***P***	**Influent**	**Digester**	***P***	**Influent**	**Digester**	***P***
carbohydrate-like	151.14 ± 44.45	76.90 ± 21.12	0.000	3.57 ± 2.15	0.93 ± 0.35	0.000	386.49 ± 52.37	350.51 ± 35.54	0.108
condensed-aromatics-like	81.57 ± 43.03	102.67 ± 62.13	0.440	2.36 ± 1.06	1.35 ± 0.63	0.017	550.49 ± 34.22	487.10 ± 42.34	0.001
lignin-like	2209.57 ± 282.79	2735.10 ± 488.95	0.006	42.76 ± 5.65	41.45 ± 13.40	0.424	390.75 ± 21.49	382.38 ± 10.02	0.457
lipid-like	298.14 ± 80.76	625.29 ± 205.36	0.000	10.40 ± 5.54	20.21 ± 7.65	0.002	362.05 ± 38.52	360.74 ± 27.64	0.944
protein-like	927.43 ± 120.60	873.29 ± 150.62	0.271	23.99 ± 1.61	15.07 ± 3.11	0.000	381.24 ± 39.08	386.48 ± 17.41	0.727
tannins-like	218.00 ± 46.22	161.38 ± 37.17	0.004	3.53 ± 1.02	1.80 ± 0.58	0.000	363.81 ± 19.69	335.63 ± 16.70	0.000
unsaturated-hydrocarbons-like	463.14 ± 98.82	646.17 ± 151.01	0.004	11.53 ± 4.71	18.64 ± 6.60	0.007	351.38 ± 28.22	369.22 ± 17.91	0.065
unclassified	139.00 ± 56.02	60.36 ± 30.99	0.000	1.87 ± 1.10	0.55 ± 0.30	0.000	619.25 ± 58.72	554.05 ± 35.33	0.004

Table S4 Relative abundance of molecular categories across different regions. Statistical significance was assessed using the Wilcoxon test, different letters indicate significant differences (*P* < 0.05).

Category	Relative abundance (%)						
	**BJ**	**CS**	**FS**	**JZ**	**QH**	**QQ**	**WZ**
carbohydrate-like	0.80 ± 0.31 bc	1.32 ± 0.26 a	1.01 ± 0.46 ab	0.88 ± 0.18 b	0.88 ± 0.09 b	1.10 ± 0.35 ab	0.53 ± 0.09 c
condensed-aromatics-like	1.53 ± 0.42 ab	1.51 ± 0.26 a	1.05 ± 0.32 b	1.74 ± 0.79 ab	1.12 ± 0.30 ab	1.50 ± 0.76 ab	1.02 ± 1.04 ab
lignin-like	47.37 ± 3.13 b	36.77 ± 4.74 c	34.32 ± 8.36 cd	29.09 ± 4.98 d	29.66 ± 3.07 d	45.55 ± 4.45 b	67.40 ± 2.96 a
lipid-like	15.69 ± 2.13 d	21.14 ± 2.62 b	25.34 ± 7.47 abc	27.42 ± 4.11 a	27.39 ± 4.44 a	15.96 ± 2.71 cd	8.50 ± 1.01 e
protein-like	15.96 ± 1.40 a	16.63 ± 4.53 ab	14.64 ± 2.97 abc	11.17 ± 2.41 c	16.31 ± 1.54 a	17.31 ± 1.64 a	13.49 ± 1.91 bc
tannins-like	2.07 ± 0.64 ab	1.89 ± 0.34 a	1.96 ± 0.86 abc	1.44 ± 0.31 bc	1.27 ± 0.22 c	2.12 ± 0.63 ab	1.86 ± 0.37 ab
unsaturated-hydrocarbons-like	0.58 ± 0.24 a	0.50 ± 0.18 a	0.63 ± 0.23 a	0.70 ± 0.34 a	0.70 ± 0.46 a	0.51 ± 0.24 a	0.20 ± 0.08 b
unclassified	15.99 ± 1.53 c	20.25 ± 2.30 b	21.05 ± 4.63 bc	27.56 ± 3.11 a	22.67 ± 1.98 b	15.94 ± 3.10 c	7.00 ± 0.76 d

Table S5 Parameters for the segmented linear regression model were fitted based on molecular weight-based segmental dissimilarity. Starting from a simple linear regression model, change points were gradually added, ultimately leading to the optimal model.

**Module**		**Number of break-point**	**R^2**	**AIC**
Digester	Original model	0	0.571	-81.65
	Adjusted model 1	1	0.931	-320.19
	Adjusted model 2	2	0.945	-347.26
	Adjusted model 3	3	0.994	-636.45
	Adjusted model 4	4	0.995	-643.99

Table S6 Influence of microbial composition and feedstock characteristics on DOM composition in anaerobic digesters. Mantel tests based on Spearman correlation were performed to individually assess the relationships between DOM composition and microbial community structure, and between DOM composition and feedstock molecular composition. To simultaneously account for the influence of both variables, multiple regression on matrices (MRM) was employed. Variation partitioning analysis (VPA) was conducted based on the MRM results to quantify the proportion of explained variance. The explained variance attributed to each individual factor includes both its unique contribution and the shared variance explained jointly with the other factor.

**Variable**	**Mantel test**		**MRM**	**VPA**
	**r**	***P***	***P***	**Explained variance (%)**
Prokaryotic community	0.28	0.001	0.035	16.85
Original substance	0.51	0.001	0.000	31.84

Table S7 Global network properties of city-specific microbial-DOM bipartite networks. '***' means statistical significance ($P < 0.001$) between empirical network and 100 randomized networks based on one-sample Student's t test.

Global properties	BJ	CS	FS	JZ	QH	QQ	WZ
Network size							
No. microbes	223	214	237	272	155	220	267
No. molecules	1679	1440	3092	1642	1915	1429	1418
Total links	19174	17853	53992	21205	19010	11771	32602
Average Degree	10.081	10.79	16.22	11.08	9.18	7.14	19.35
No. of modules (simulated annealing)	5	5	3	4	4	4	4
Nestedness							
Empirical network	3.73 ***	6.36 ***	3.39 ***	4.35 ***	2.86 ***	3.86 ***	8.40 ***
Randomized network (n = 100)	5.53 ± 0.059	8.20 ± 0.066	4.41 ± 0.032	5.92 ± 0.046	3.88 ± 0.052	5.74 ± 0.060	7.93 ± 0.045
Weighted nestedness							
Empirical network	0.52 ***	0.41 ***	0.68 ***	0.45 ***	0.67 ***	0.45 ***	0.32 ***
Randomized network (n = 100)	0.53 ± 0.0054	0.44 ± 0.0054	0.72 ± 0.0024	0.47 ± 0.0046	0.72 ± 0.0038	0.42 ± 0.0055	0.49 ± 0.0049

Supplementary Figures

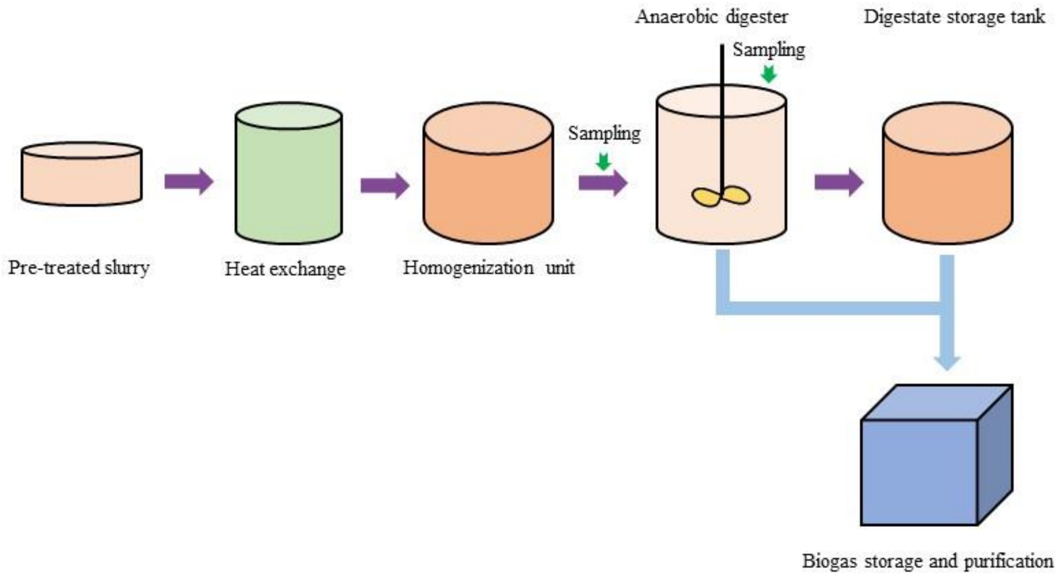

Fig. S1 Process flow of the anaerobic digestion system in this study.

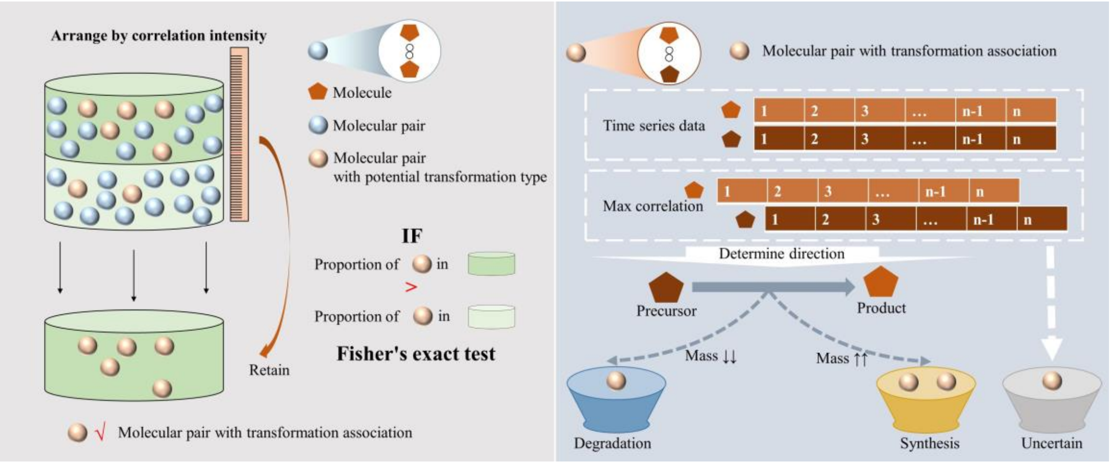

Fig. S2 Framework for the identification and analysis of molecular transformations. Potential molecular pairs with transformation relationships were identified and categorized as degradation, synthesis, or uncertain, with precursor and product identities assigned for degradation and synthesis transformations.

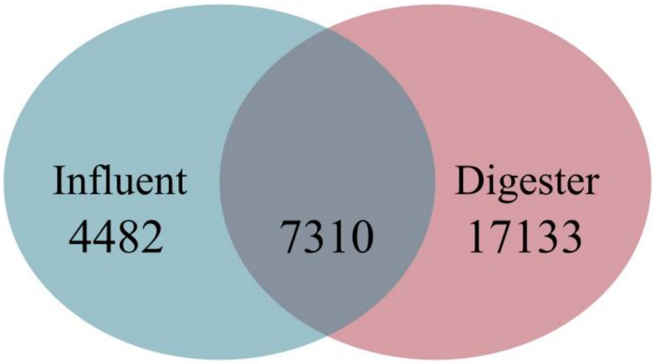

Fig. S3 Number of identified molecules in influent and the digester samples.

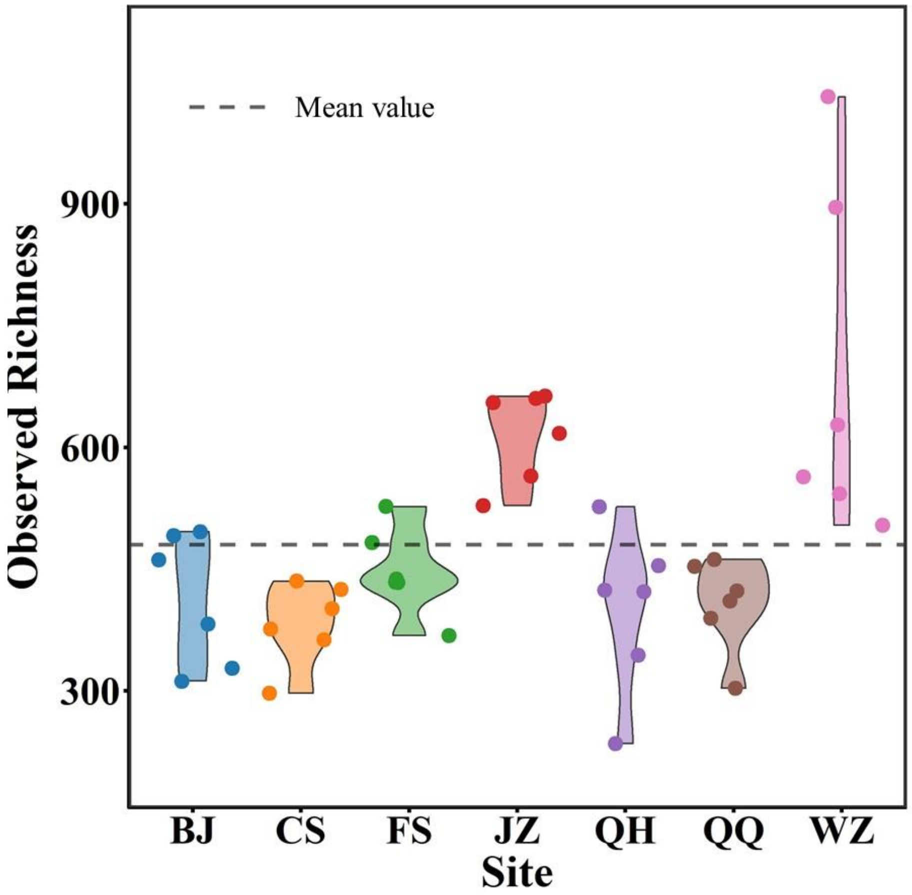

Fig. S4 Microbial observed richness in the digester samples. Observed richness, expressed as the number of detected ZOTUs, averaged 479.83 ± 150.15 per sample.

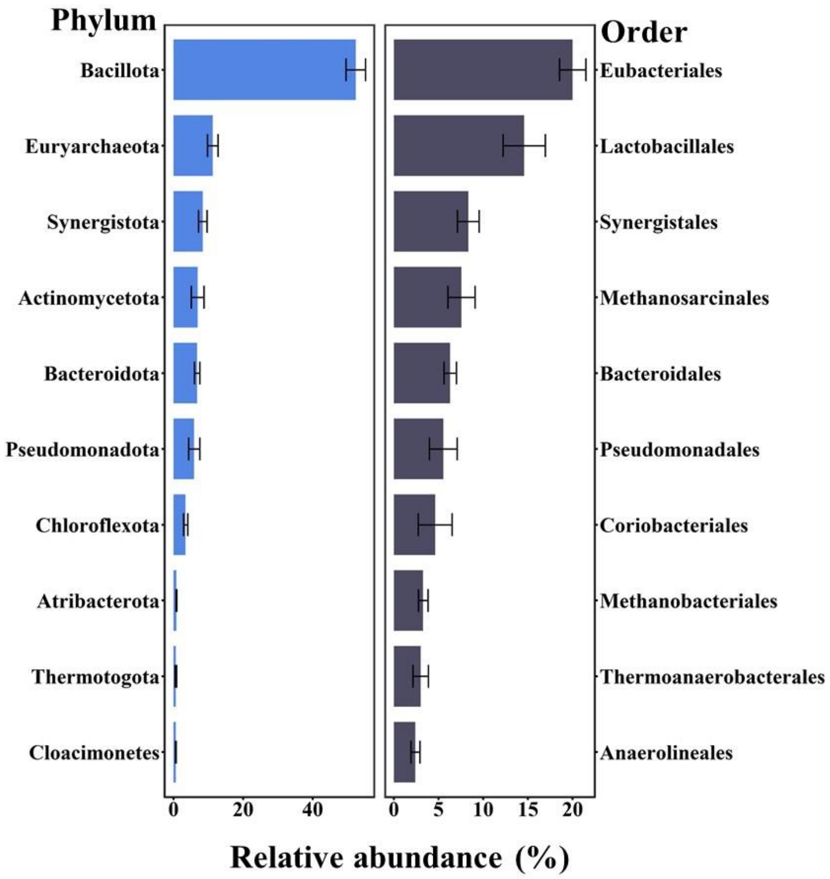

Fig. S5 Taxonomic composition of abundant microorganisms in the anaerobic digesters. The 10 most abundant taxa at the phylum and order levels were determined based on mean relative abundance across all samples. Bars show mean values， and error bars denote standard errors.

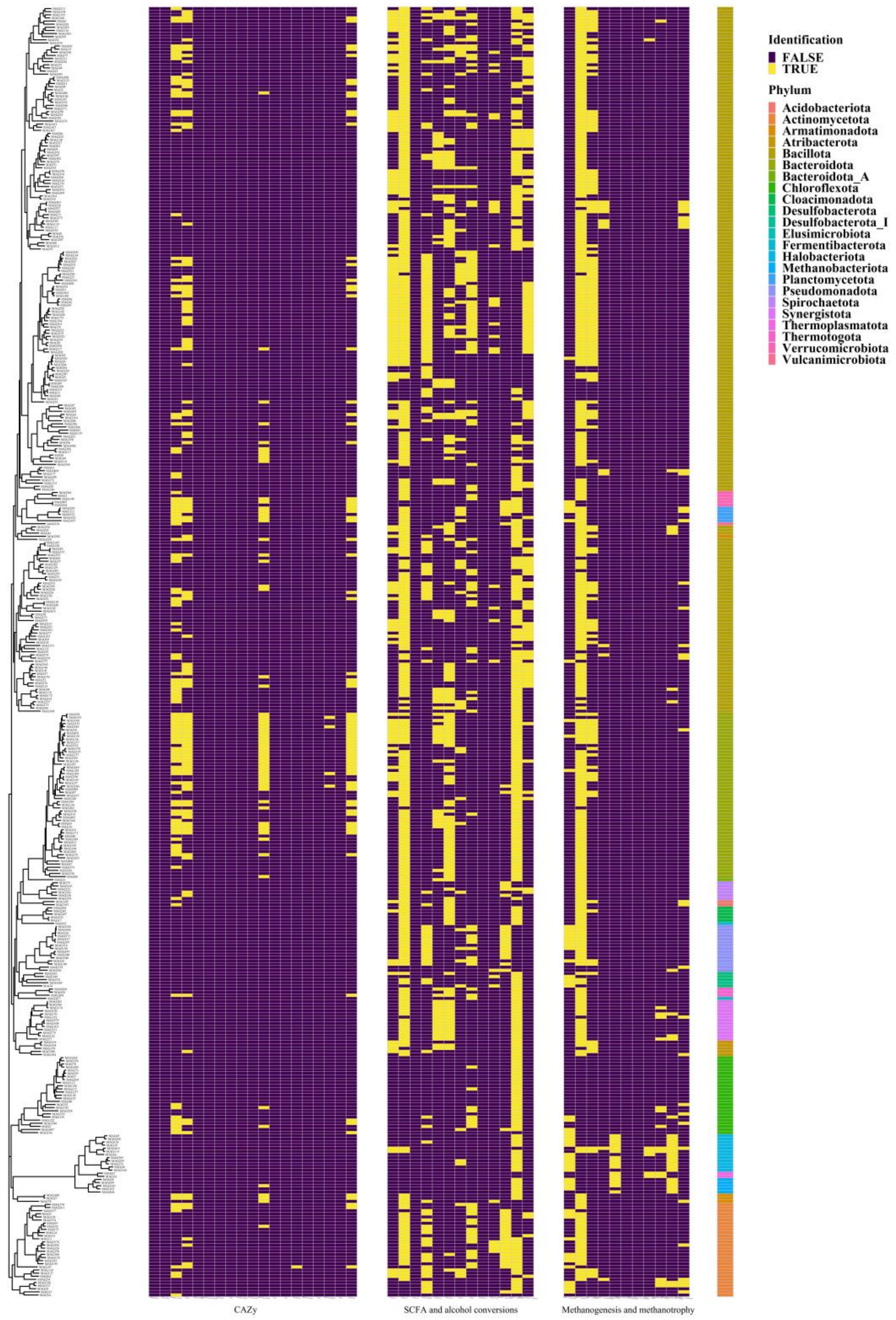

Fig. S6 Metabolic functional annotations of major microbial taxa. A total of 413 MAGs assembled from metagenomes were taxonomically classified and annotated to determine whether they encoded specific carbon-metabolism capabilities.

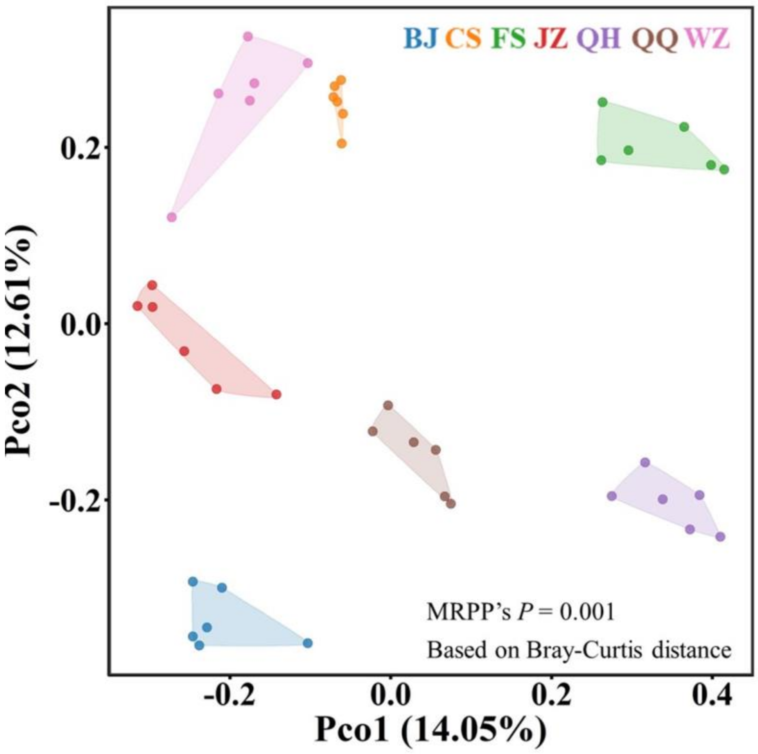

Fig. S7 The dissimilarity in microbial community composition among samples was assessed based on Bray-Curtis distances. Dimensionality reduction was performed using principal coordinates analysis (PCoA) to aid interpretation. The statistical significance of compositional differences among groups was evaluated using the multi-response permutation procedure (MRPP).

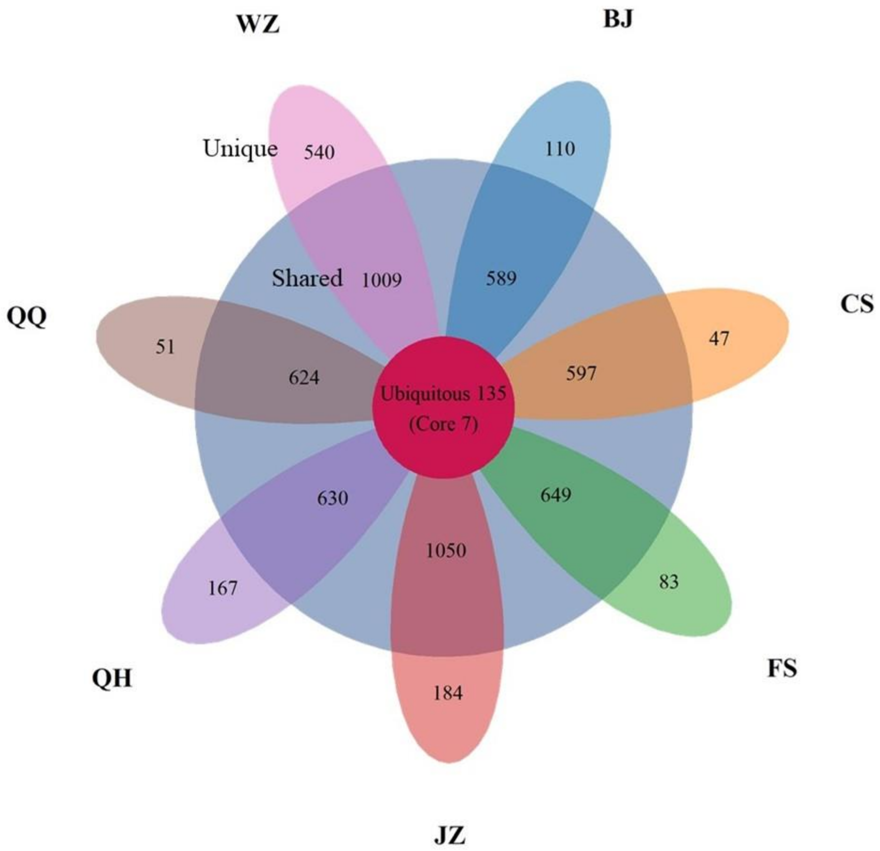

Fig. S8 Counts of shared microbes in digesters. Unique ZOTUs were detected exclusively in a single region. Shared ZOTUs were detected in multiple regions. Ubiquitous ZOTUs were detected across all regions, among which 7 were found in all samples.

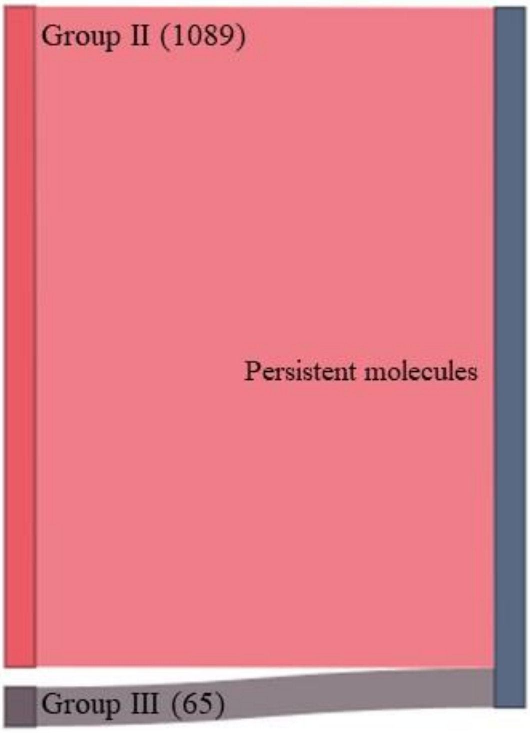

Fig. S9 Correspondence between molecular groups distinguished by segmental dissimilarity fitting results and molecules identified as persistent across all samples. Nearly all of the persistent molecules belong to Group II (1,089/1,154), accounting for 94.37%, while a small number belong to Group III, with 65 molecules.

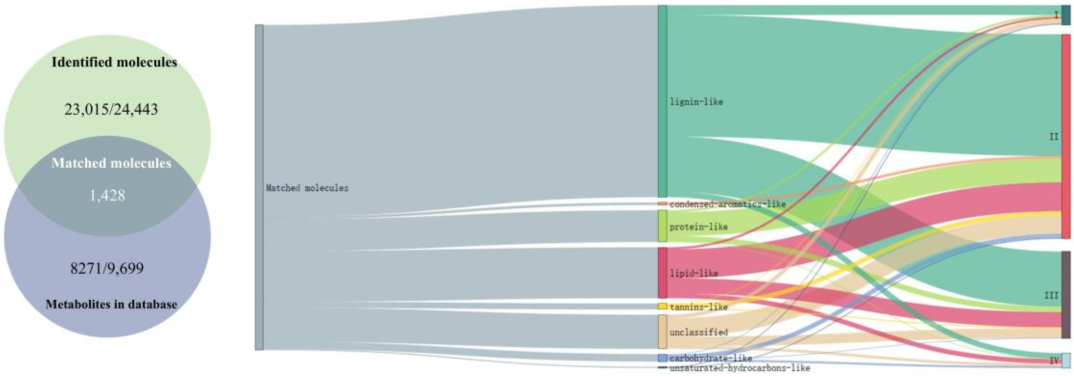

Fig. S10 Comparison between identified molecules and metabolite datasets.

(A) Molecules identified in anaerobic digesters were matched to metabolite datasets based on molecular formulae, yielding a total of 1,428 matched molecules. (B) Classification of the identified molecules according to element-based natural organic matter-like categories and the molecular weight intervals defined in the manuscript.

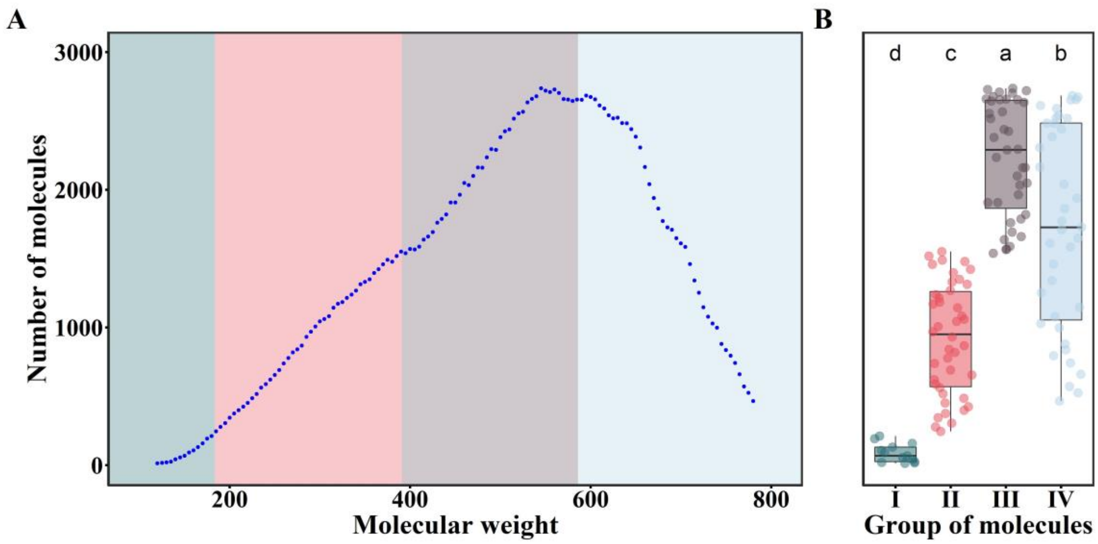

Fig. S11 The number of detected molecules across different molecular weights. The results were generated using a sliding window approach with a window width of 40 and a step size of 5; panel (A) covers all intervals, and panel (B) corresponds to the four identified groups. Different letters in (B) indicate significant differences ($P <$ 0.05) based on the Wilcoxon test.

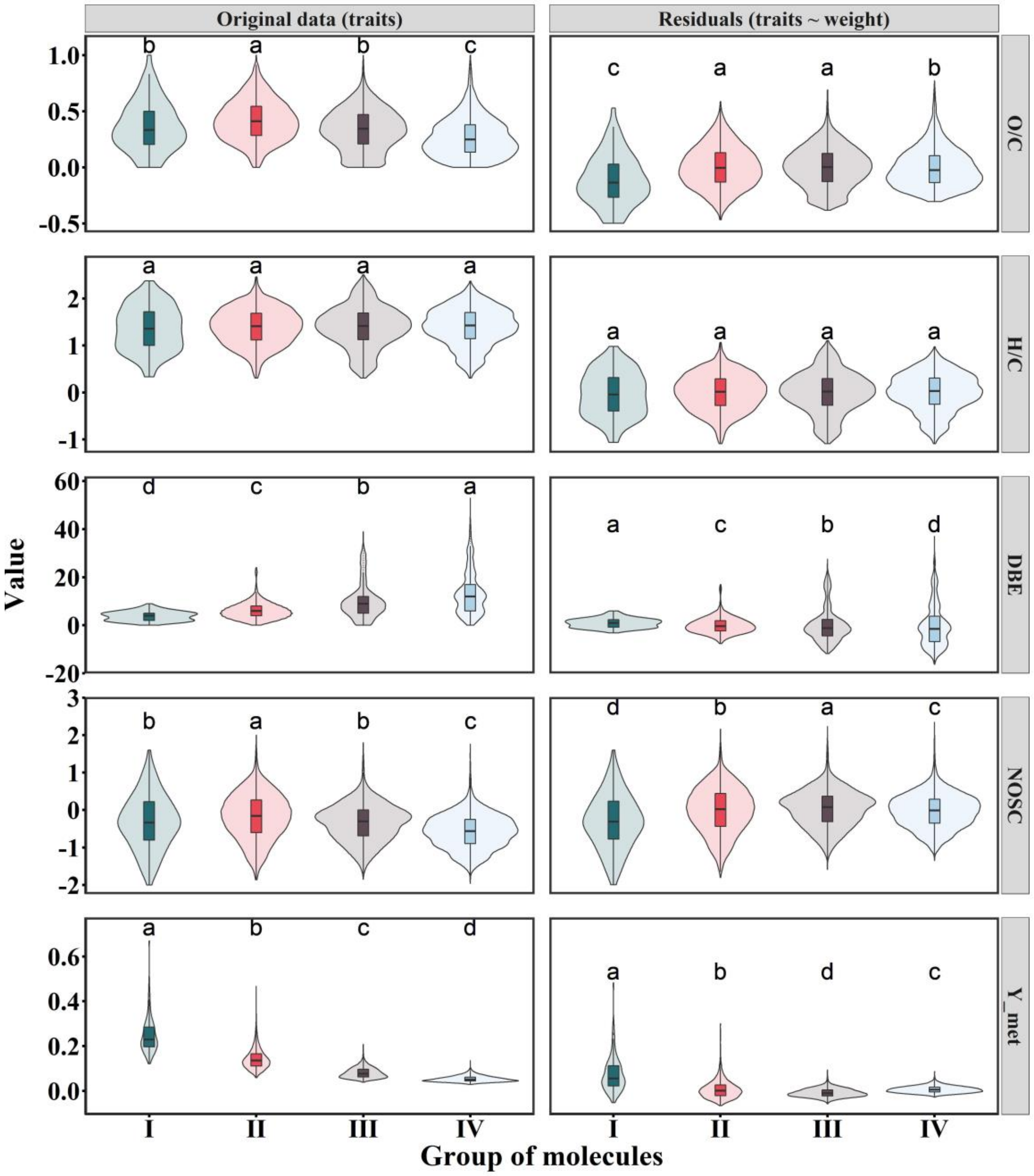

Fig. S12 Comparison of molecular traits across different molecular groups. To eliminate the influence of molecular weight, in addition to comparing the original data of molecular traits, the residuals from the linear fit based on molecular weight were also compared. Different letters indicate significant differences ($P < 0.05$) based on the Wilcoxon test.

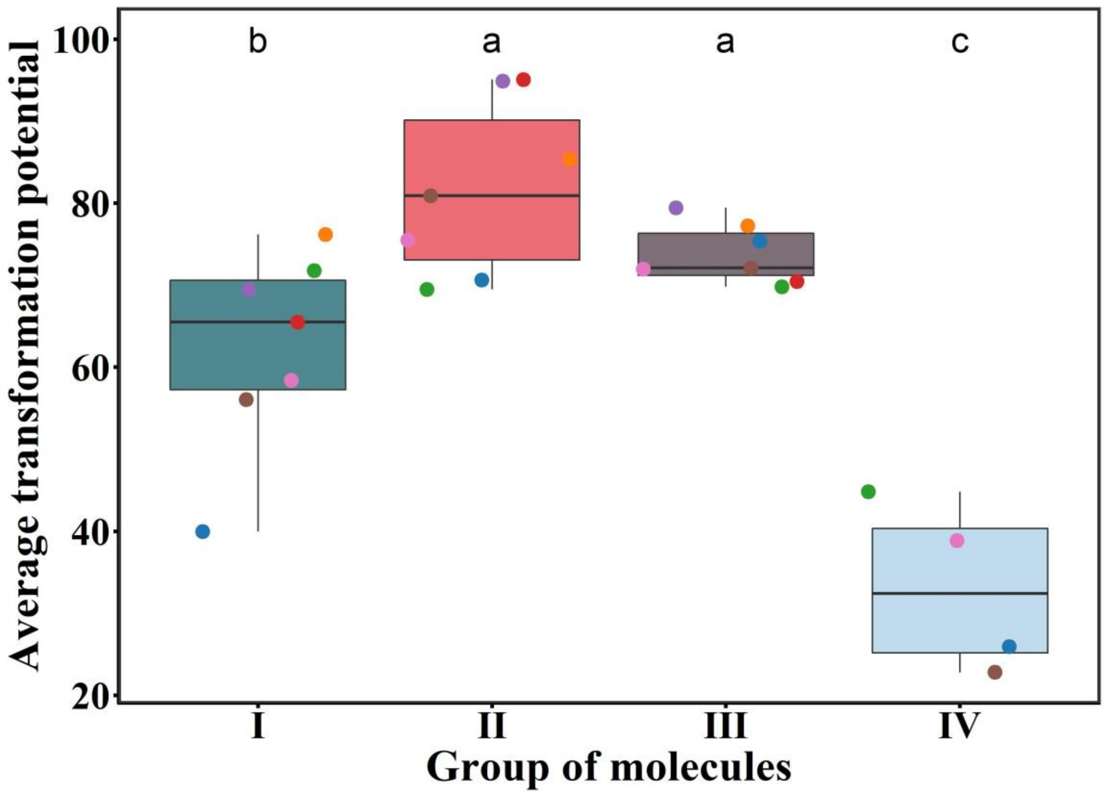

Fig. S13 Average transformation potential of molecules with different molecular weight range. The transformation potential of a molecule was defined by the number of valid transformation-related molecular pairs in which it was identified. In the visualization, each point represents the mean transformation potential of molecules within each group. Different letters indicate significant differences (*P* < 0.05) based on the Wilcoxon test.

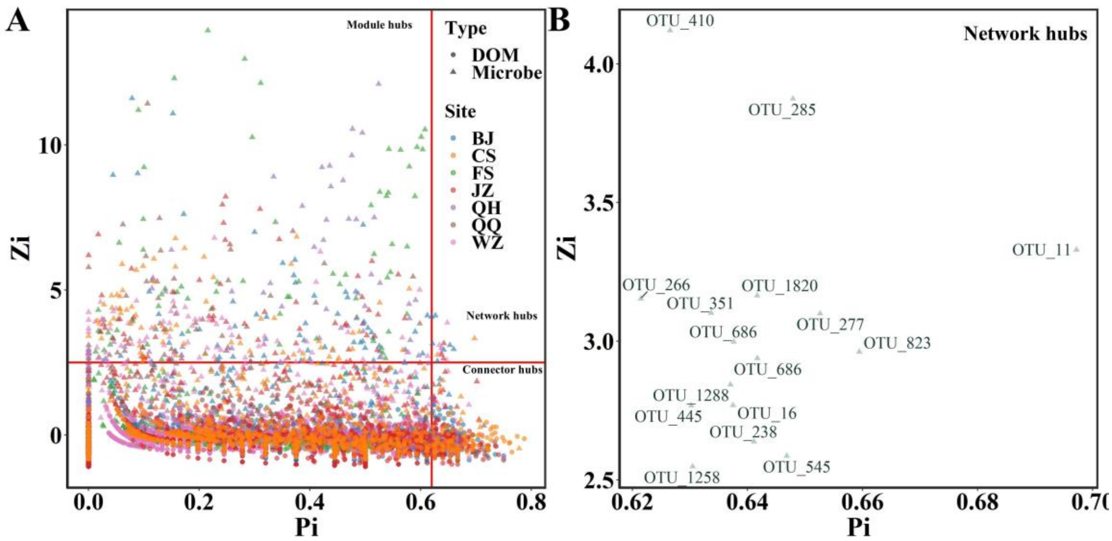

Fig. S14 Key microbes and molecules identified from microbial-DOM bipartite networks. (A) Roles of microbes and molecules within the network. Key roles include network hubs, module hubs, and connector hubs, identified based on their contributions to within-module connectivity and cross-module linkages. (B) All microbes identified as network hubs.

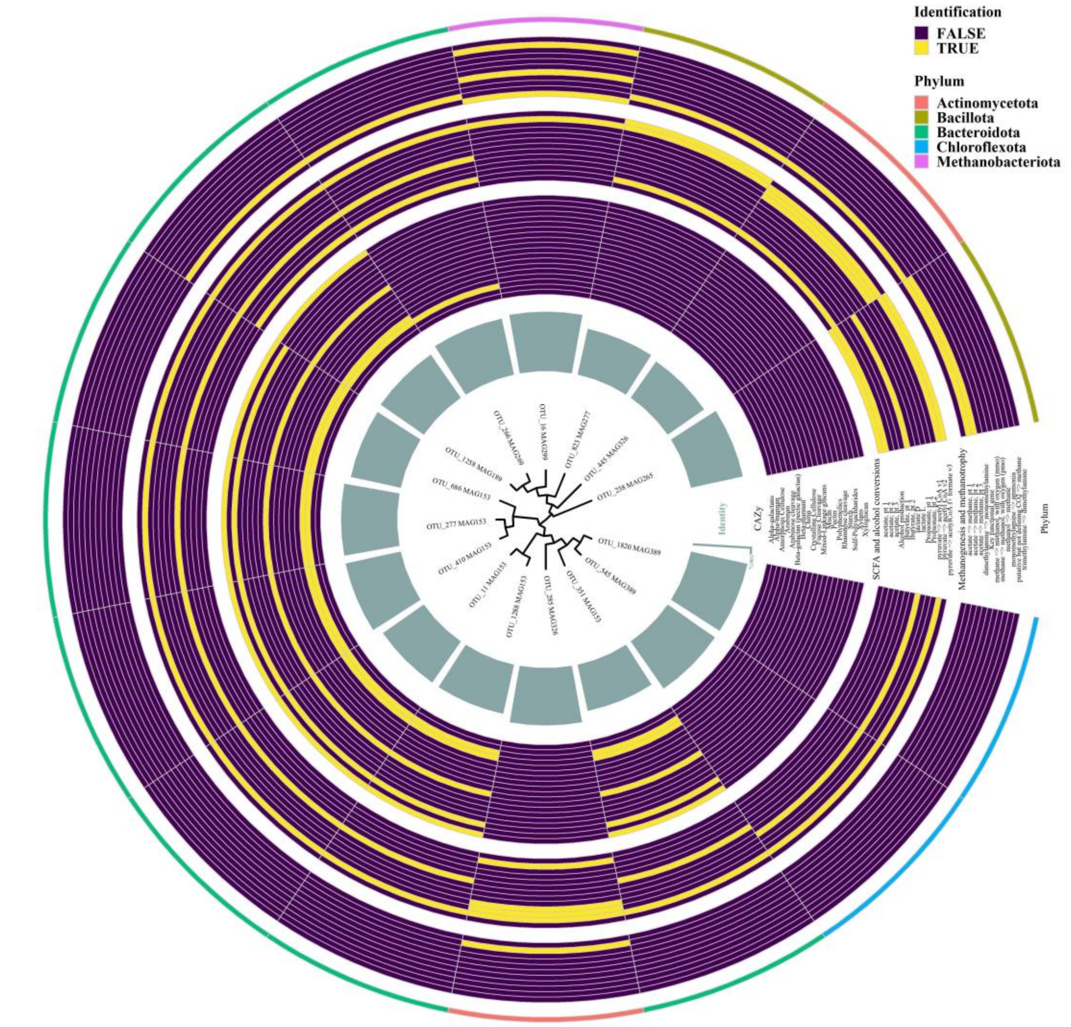

Fig. S15 Carbon metabolic capacities of microbes identified as network hubs. Microbial taxa identified as network hubs from the microbial-DOM bipartite networks are shown. The 16S rRNA gene sequences obtained from amplicon sequencing were matched to 16S rRNA sequences recovered from MAGs. Bar plots indicate sequence similarity between amplicon-derived and MAG-associated 16S rRNA sequences. The heatmap summarizes the inferred carbon metabolic capacities of these microbes together with their phylogenetic classifications at Phylum level.

Reference

1. Hu J, Liu CG, Zhang WK, Liu XW, Dong B, Wang ZD *et al.* Decomposing the molecular complexity and transformation of dissolved organic matter for innovative anaerobic bioprocessing. Nat. Commun. 2025;16:4859.
2. Wu L, Ning D, Zhang B, Li Y, Zhang P, Shan X *et al.* Global diversity and biogeography of bacterial communities in wastewater treatment plants. Nat. Microbiol. 2019;4:1183-1195.
3. Yang X, Zhang Z, Li S, He Q, Peng X, Du X *et al.* Fungal dynamics and potential functions during anaerobic digestion of food waste. Environ. Res. 2022;10.1016/j.envres.2022.113298113298.
4. Dreywood R. Qualitative test for carbohydrate material. Ind. Eng. Chem. Anal. Ed. 1946;18:499.
5. Smith PK, Krohn RI, Hermanson GT, Mallia AK, Gartner FH, Provenzano MD *et al.* Measurement of protein using bicinchoninic acid. Anal. Biochem. 1985;150:76-85.
6. Du X, Gu S, Zhang Z, Li S, Zhou Y, Zhang Z *et al.* Spatial distribution patterns across multiple microbial taxonomic groups. Environ. Res. 2023;223:115470.
7. Yang X, Feng K, Wang S, Yuan MM, Peng X, He Q *et al.* Unveiling the deterministic dynamics of microbial meta-metabolism: a multi-omics investigation of anaerobic biodegradation. Microbiome. 2024;12:166.
8. Feng K, Wang S, He Q, Bonkowski M, Bahram M, Yergeau E *et al.* CoBacFM: Core bacteria forecast model for global grassland pH dynamics under future climate warming scenarios. One Earth. 2024;10.1016/j.oneear.2024.06.002.
9. Yang X, Feng K, Wang S, Yuan MM, Peng X, He Q *et al.* Unveiling the deterministic dynamics of microbial meta-metabolism: a multi-omics investigation of anaerobic biodegradation. Microbiome. 2024;12.
10. Edgar RC. UNOISE2: Improved Error-Correction for Illumina 16s and ITS Amplicon Sequencing. bioRxiv. 2016;http://doi.org/10.1101/081257http://doi.org/10.1101/081257.
11. Wang Q, Garrity GM, Tiedje JM, Cole JR. Naive Bayesian classifier for rapid

assignment of rRNA sequences into the new bacterial taxonomy. Appl. Environ. Microbiol. 2007;73:5261-5267.

12. Xu H, Luo X, Qian J, Pang X, Song J, Qian G *et al.* FastUniq: a fast de novo duplicates removal tool for paired short reads. PLoS One. 2012;7:e52249.

13. Bolger AM, Lohse M, Usadel B. Trimmomatic: a flexible trimmer for Illumina sequence data. Bioinformatics. 2014;30:2114-2120.

14. Ewels P, Magnusson M, Lundin S, Käller M. MultiQC: summarize analysis results for multiple tools and samples in a single report. Bioinformatics. 2016;32:3047-3048.

15. Uritskiy GV, DiRuggiero J, Taylor J. MetaWRAP-a flexible pipeline for genome-resolved metagenomic data analysis. Microbiome. 2018;6:158.

16. Olm MR, Brown CT, Brooks B, Banfield JF. dRep: a tool for fast and accurate genomic comparisons that enables improved genome recovery from metagenomes through de-replication. ISME J. 2017;11:2864-2868.

17. Chaumeil PA, Mussig AJ, Hugenholtz P, Parks DH. GTDB-Tk: a toolkit to classify genomes with the Genome Taxonomy Database. Bioinformatics. 2019;36:1925-1927.

18. Shaffer M, Borton MA, Bolduc B, Faria JP, Flynn RM, Ghadermazi P *et al.* kb_DRAM: annotation and metabolic profiling of genomes with DRAM in KBase. Bioinformatics. 2023;39.

19. Shaffer M, Borton MA, McGivern BB, Zayed AA, La Rosa SL, Solden LM *et al.* DRAM for distilling microbial metabolism to automate the curation of microbiome function. Nucleic Acids Res. 2020;48:8883-8900.

20. Yu S, Lv J, Jiang L, Geng P, Cao D, Wang Y. Changes of Soil Dissolved Organic Matter and Its Relationship with Microbial Community along the Hailuogou Glacier Forefield Chronosequence. Environ. Sci. Technol. 2023;57:4027-4038.

21. Liu M, Graham N, Gregory J, Elimelech M, Yu W. Towards a molecular-scale theory for the removal of natural organic matter by coagulation with trivalent metals. Nat. Water. 2024;2:285-294.

22. Hu J, Kang L, Li Z, Feng X, Liang C, Wu Z *et al.* Photo-produced aromatic

compounds stimulate microbial degradation of dissolved organic carbon in thermokarst lakes. Nat. Commun. 2023;14:3681.

23. Singer GA, Fasching C, Wilhelm L, Niggemann J, Steier P, Dittmar T *et al.* Biogeochemically diverse organic matter in Alpine glaciers and its downstream fate. Nat. Geosci. 2012;5:710-714.

24. Caspi R, Billington R, Fulcher CA, Keseler IM, Kothari A, Krummenacker M *et al.* The MetaCyc database of metabolic pathways and enzymes. Nucleic Acids Res. 2018;46:D633-D639.

25. Reshef DN, Reshef YA, Finucane HK, Grossman SR, McVean G, Turnbaugh PJ *et al.* Detecting novel associations in large data sets. Science. 2011;334:1518-1524.

26. Deng Y, Zhang P, Qin Y, Tu Q, Yang Y, He Z *et al.* Network succession reveals the importance of competition in response to emulsified vegetable oil amendment for uranium bioremediation. Environ. Microbiol. 2016;18:205-218.

27. Xia M, Li P, Liu J, Qin W, Dai Q, Wu M *et al.* Long-term fertilization promotes the microbial-mediated transformation of soil dissolved organic matter. Commun. Earth Environ. 2025;6:114.

28. Peng X, Feng K, Yang X, He Q, Zhao B, Li T *et al.* iNAP 2.0: Harnessing metabolic complementarity in microbial network analysis. iMeta. 2024;10.1002/imt2.235.

29. Hu A, Choi M, Tanentzap AJ, Liu J, Jang KS, Lennon JT *et al.* Ecological networks of dissolved organic matter and microorganisms under global change. Nat. Commun. 2022;13:3600.

30. Deng Y, Jiang Y-H, Yang Y, He Z, Luo F, Zhou J. Molecular ecological network analyses. BMC Bioinformatics. 2012;13:113.

31. Feng K, Peng X, Zhang Z, Gu S, He Q, Shen W *et al.* iNAP: An integrated network analysis pipeline for microbiome studies. iMeta. 2022;1.
